# Micro/Nano-Reconfigurable Robots for Intelligent Carbon Management in Confined-Space Life-Support Systems

**DOI:** 10.1007/s40820-025-01932-9

**Published:** 2025-11-04

**Authors:** Wei Lu, Rimei Chen, Lianlong Zhan, Qin Xiang, Renting Huang, Lei Wang, Shuangfei Wang, Hui He

**Affiliations:** Guangxi Key Laboratory of Clean Pulp and Papermaking and Pollution Control, School of Light Industry and Food Engineering, Guangxi University, Nanning, 530004 People’s Republic of China

**Keywords:** Micro/nano, Reconfigurable, Robot, Confined space, CO_2_ management, Efficient regeneration

## Abstract

Strategically coupling nanoparticle hybrids and internal thermosensitive molecular switches establishes an innovative paradigm for constructing micro/nanoscale-reconfigurable robots, facilitating energy-efficient CO_2_ management in life-support systems of confined space. Here, a micro/nano-reconfigurable robot is constructed from the CO_2_ molecular hunters, temperature-sensitive molecular switch, solar photothermal conversion, and magnetically-driven function engines. The molecular hunters within the molecular extension state can capture 6.19 mmol g^−1^ of CO_2_ to form carbamic acid and ammonium bicarbonate. Interestingly, the molecular switch of the robot activates a molecular curling state that facilitates CO_2_ release through nano-reconfiguration, which is mediated by the temperature-sensitive curling of Pluronic F127 molecular chains during the photothermal desorption. Nano-reconfiguration of robot alters the amino microenvironment, including increasing surface electrostatic potential of the amino group and decreasing overall lowest unoccupied molecular orbital energy level. This weakened the nucleophilic attack ability of the amino group toward the adsorption product derivatives, thereby inhibiting the side reactions that generate hard-to-decompose urea structures, achieving the lowest regeneration temperature of 55 °C reported to date. The engine of the robot possesses non-contact magnetically-driven micro-reconfiguration capability to achieve efficient photothermal regeneration while avoiding local overheating. Notably, the robot successfully prolonged the survival time of mice in the sealed container by up to 54.61%, effectively addressing the issue of carbon suffocation in confined spaces. This work significantly enhances life-support systems for deep-space exploration, while stimulating innovations in sustainable carbon management technologies for terrestrial extreme environments.

**Supplementary Information:**

The online version contains supplementary material available at 10.1007/s40820-025-01932-9.

## Highlights

- The micro/nano-reconfigurable robots for life-support systems were fabricated by CO₂-capturing molecular hunters, temperature-sensitive molecular switches, and solar photothermal conversion/magnetically-driven dual function engines.
- The ultralow regeneration temperature (55 °C) and non-contact heat management of robots were achieved through nano-reconfiguration of internal temperature-responsive molecules and micro-reconfiguration of magnetic/photothermal synergy of Fe_3_O_4_ nanoparticles.
- Exceptional dynamic carbon management of robots extended the survival time of mice in confined spaces by 54.61%.

**Supplementary Information:**

The online version contains supplementary material available at 10.1007/s40820-025-01932-9.

## Introduction

The scavenge of CO_2_ generated continuously by crew respiration, material oxidation, and equipment operation in confined environments (e.g., submarines, manned spacecraft, and space stations) is recognized as a critical process in environmental control and life-support systems (ECLSS) to prevent life-threatening risks caused by CO_2_ accumulation [1–5]. However, the challenge of CO_2_ removal in ECLSS is exacerbated by low concentrations of CO_2_, the weak adsorption driving forces, and stringent energy requirements for material regeneration [6]. Current methods to address this problem primarily rely on liquid amines, which require relatively high energy for regeneration and may pose a health hazard due to amine leakage [7]. In contrast, physical adsorption materials such as molecular sieves [8], zeolites [9], and metal–organic frameworks (MOFs) [10, 11] have garnered significant attention due to their relatively safe operation and ease of regeneration. However, the inherent hydrophilicity of their skeletons requires complex and expensive hydrophobic modification processes to improve the practicality [12].

Conventional amino-functionalized CO_2_ removal materials utilize amino functional groups to chemically react with CO_2_, where primary and secondary amines form carbamic acid. Notably, the presence of water vapor in confined systems facilitates the formation of ammonium bicarbonate species via proton transfer mechanisms, thereby enhancing amine utilization efficiency and achieving CO_2_ adsorption capacities of 3–6 mmol g^−1^ [13–15]. However, the strong chemical between amine groups and CO_2_ necessitates elevated energy input during desorption. Particularly concerning is the potential evolution of adsorbed products into urea derivatives with more stable configurations, requiring higher decomposition temperatures (typically above 110 °C) and consequently increasing regeneration energy consumption [16, 17]. Over a decade ago, pioneering work by Hoshino’s group [18] demonstrated the potential of thermoresponsive materials for CO_2_ capture applications, where temperature-induced phase transitions facilitate sorbent regeneration at reduced temperatures. Building upon this conceptual breakthrough, our research team has successfully engineered a series of temperature-sensitive fiber-based solid amine adsorbents exhibiting exceptional CO_2_ capture capacity (> 6 mmol g^−1^) coupled with ultralow regeneration temperature at 60 °C, representing one of the class-leading energy-efficient regeneration temperatures reported to date for comparable materials [19–22]. However, the mechanisms of thermosensitive properties in low-temperature regeneration processes, particularly their specific modulatory effects on material regeneration pathways, remain inadequately understood. Our systematic investigations aim to unravel these intricate dynamic interactions at the molecular level, with the ultimate goal of achieving breakthroughs in intelligent material regeneration technologies.

Another strategy to further reduce the regenerative energy consumption of the carbon capture process is to use clean energy sources such as solar power [23]. Various materials, including activated carbon [24], polydopamine [25], and graphene with π-π conjugated structures [26], as well as metal nano-structures, have been proposed for the photothermal desorption of CO_2._ For example, Li et al. [27] achieved a CO_2_ adsorption capacity of 1.14 mmol g^−1^ by incorporating Ag nano-crystals into the metal–organic framework UiO-66 (Ag/UiO-66), releasing up to 90.5% of the CO_2_ through photothermal localized heating. This “in situ” heating approach, which utilizes low-cost, high-energy solar power, demonstrates potential to reduce regeneration energy consumption compared to conventional external temperature-swing desorption methods. However, current photothermally regenerative carbon capture materials suffer from adaptive thermal management deficiencies, where the energy input/output process is limited by a unidirectional photothermal conversion mechanism, making it difficult to dynamically regulate the temperature field distribution [28]. In particular, the single-minded pursuit of maximizing the photothermal conversion efficiency may overlook the critical challenges posed by localized thermal accumulation under continuous high-intensity sunlight irradiation, e.g., prolonged exposure of materials to high temperatures may lead to thermal oxidative deactivation, thermal CO_2_-induced deactivation, and volatilization/degradation to accelerate the aging process, thereby significantly reducing service life [29–32]. Fortunately, the movable Fe_3_O_4_ nanoparticles (Fe_3_O_4_ NPs) with photothermal conversion and magnetic field actuation have the potential to function as magnetically actuated robots, providing an innovative solution for non-contact thermal management [33, 34].

**Fig. 1:**
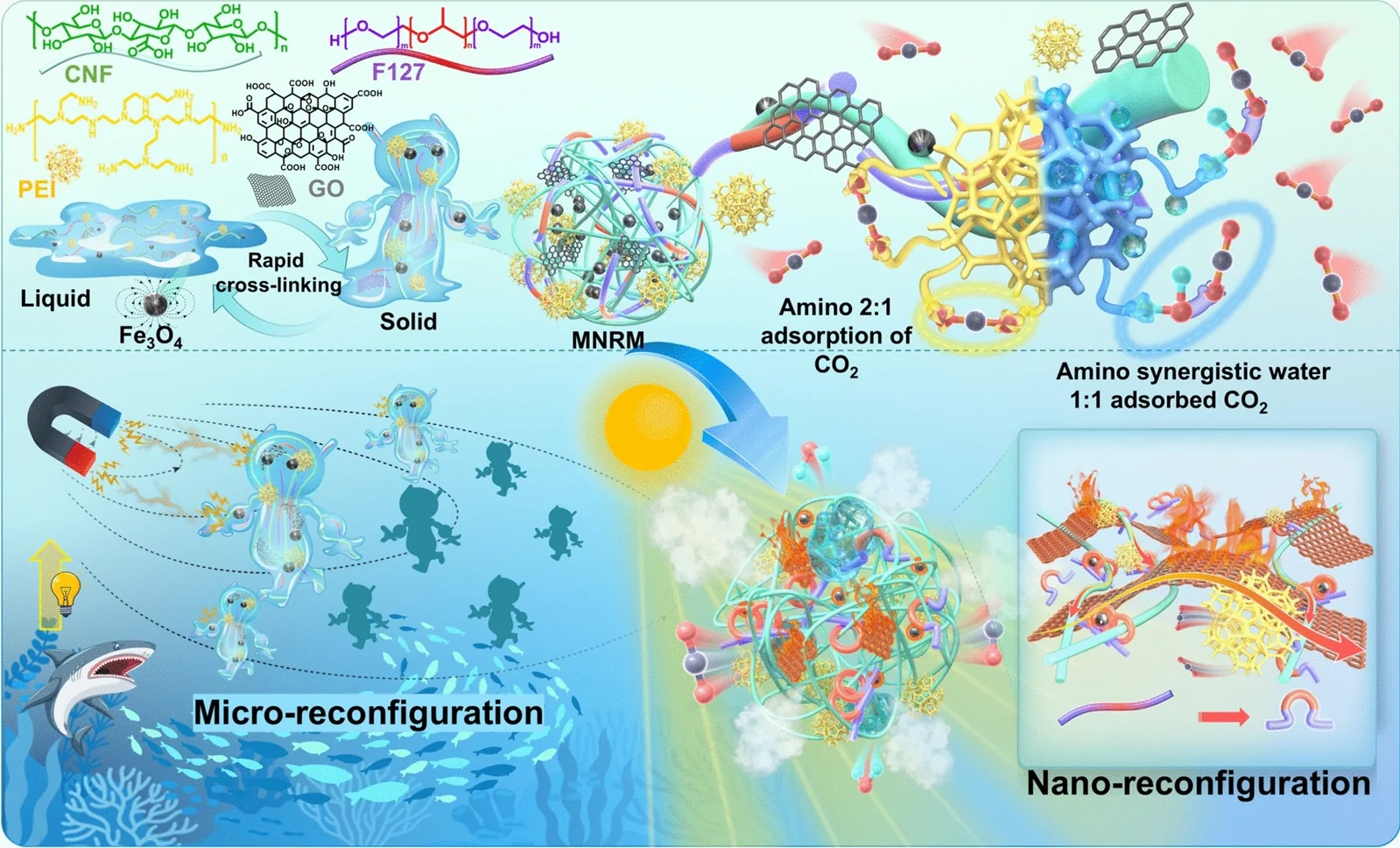
Design of MNRM with micro/nanoscale reconfiguration for energy-efficient CO_2_ capture

Inspired by the schooling behavior of fish, in which individual fish exhibit chaotic movements internally but coordinated action overall when pursued by predators, the intelligent micro/nanoscale reconfigurable robot (MNRM) breaks away from the passive properties of traditional materials and demonstrates the environmental responsiveness of life-like organisms (Fig. 1). The robot consists of cellulose nano-fibers (CNF) backbone carrying the nano-engine Fe_3_O_4_ NPs, the thermally conductive bridge graphene oxide (GO), the temperature molecular machine Pluronic F127 (F127), and polyethyleneimine (PEI). In CO_2_ scavenging missions, each robot forms a chemical “super hunter” through a high density of amino groups, generating carbamic acid and ammonium bicarbonate, which exhibit excellent CO₂ adsorption capacity (6.19 mmol g^−1^ at 25 °C). The core innovation lies in the temperature-induced molecular machinery, where the thermosensitive F127 network triggers a nanoscale conformational transition stimulated by high temperatures, driving the molecular chains from the stretched to the curled state. This molecular reconfiguration not only enhances the electrostatic potential of the amine surface, but also reduces the lowest unoccupied molecular orbital (LUMO) energy level of the system, which can interfere with the adsorption product transition and facilitate the robot's switching to low-temperature regeneration (55 °C) mode. Crucially, the constructed photothermal-magnetic motion synergistic strategy breaks through the traditional thermal management dilemma, in which the photothermal module provides the main energy input, and the magnetic motion module is remotely regulated to realize the dynamic regulation of heat and avoid local overheating. Notably, the intelligent micro/nanoscale reconfigurable robot demonstrated excellent dynamic carbon metabolism regulation in a confined-environment animal model, prolonging the survival time of mice by up to 54.61% and effectively mitigating the risk of hypercapnia-induced lung failure.

## Experimental Section

### Materials

Bagasse pulp fibers (CF) were obtained from Guangxi Guangye Sugar Co., Ltd. (Guigang, China). A single-layer graphene oxide aqueous dispersion (GO, 10 mg g^−1^) was purchased from Hangzhou Gaoxi Technology Co., Ltd. (Hangzhou, China). Pluronic F127 was sourced from Sigma-Aldrich. Polyethyleneimine (PEI, M.W. 10,000, ≥ 99%), iron oxide (II, III) (Fe_3_O_4_, 300–500 nm, 97% trace metals basis), and epichlorohydrin (ECH, AR) were purchased from Aladdin (Shanghai, China).

### Preparation of Micro/Nanoscale Reconfigurable Robots (MNRM)

The MNRM was synthesized by cross-linking CNF, F127, GO, Fe_3_O_4_ NPs, and PEI using ECH as the cross-linking agent. Specifically, a homogeneous mixture containing 10 mL of CNF suspension (0.6 wt%), 7.0 g PEI, 1 mL GO suspension (2.5 mg mL^−1^), and varying mass percentages of Fe_3_O_4_ NPs (12–22 wt%) and F127 (12–28 wt%) (calculated relative to the total dry mass of CNF, PEI, and GO) was mechanically stirred at 300 rpm. Then, the ECH was slowly added dropwise to the mixture until gelation occurred. The resulting product was washed with deionized water and freeze-dried for 48 h. The sample exhibiting optimal performance (20% and 15% mass of Fe_3_O_4_ and F127, respectively) was designated MNRM. Materials with different compositions were labeled as MNRM-Fe_3_O_4_(x)/F127(x), where x indicates the mass percentage of each component.

### Mouse Confined-Space Survival Experiment

This study evaluated the CO_2_ scavenging capabilities of MNRM in a mouse confined-space model through two independent experimental batches. The experimental model was based on a typical mouse hypoxia tolerance experiment [35]. Briefly, batch A used 7-week-old SPF-grade ICR mice (weight 31.0 ± 0.2 g, n = 3 per group), with a blank control group (no CO_2_ scavenging material) and an MNRM experimental group. Survival time was recorded, and lung tissue was collected postmortem for H&E staining and pathological analysis. Batch B used 6-week-old SPF-grade ICR mice (weight 17.9 ± 0.3 g, n = 3 per group), with three groups: blank control, MNRM experimental group, and MOF reference group. Changes in gas composition inside the bottles were recorded using a headspace analyzer. Detailed parameter settings can be found in the supporting information.

## Results and Discussion

### Preparation and Microreconfiguration Properties of MNRM

**Fig. 2:**
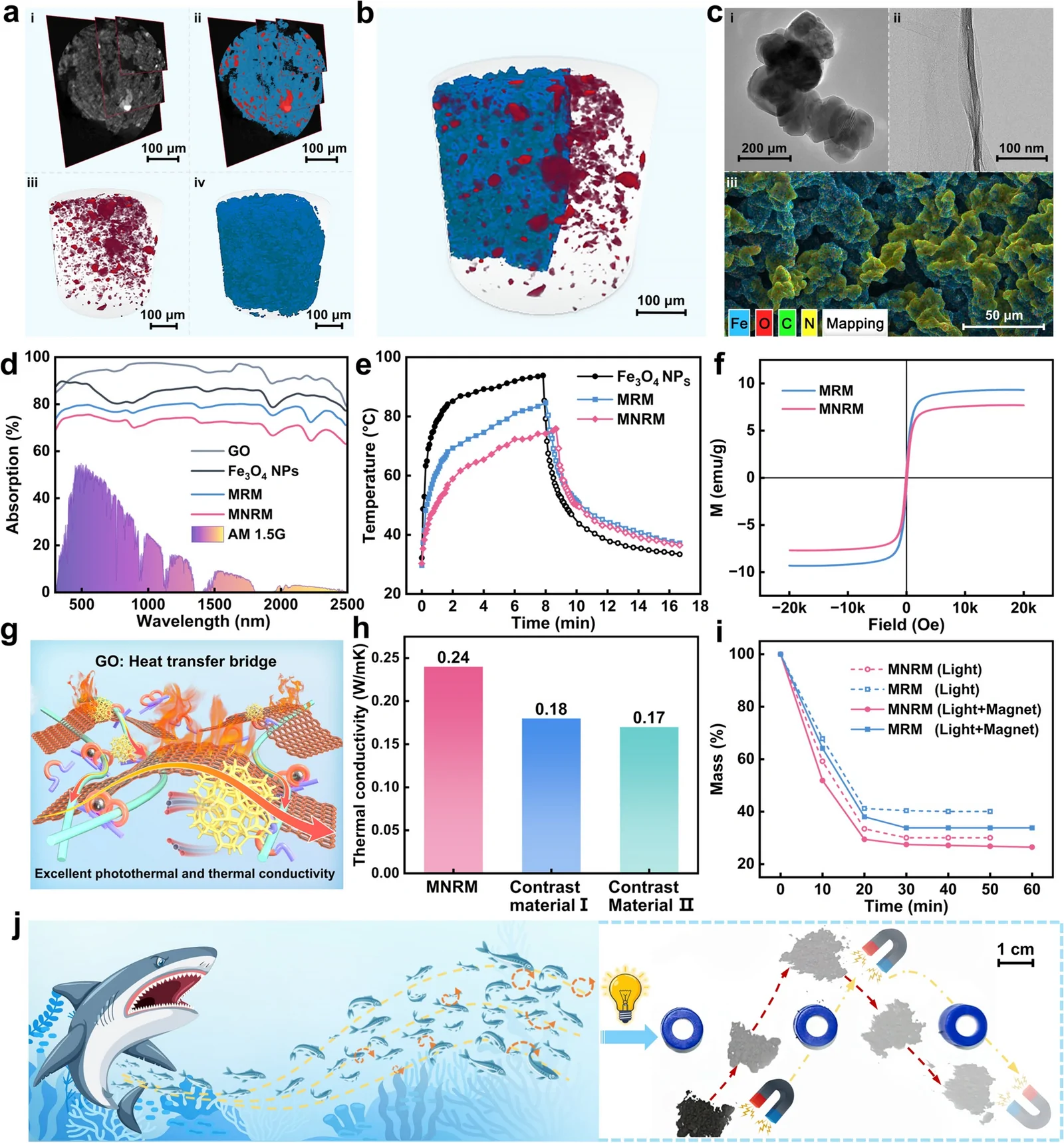
Preparation and micro-reconfiguration properties of MNRM. **a** Nano-CT characterization of MNRM: (i) 2D slice view, (ii) color-coded reconstruction 2D slice, and 3D rendered images of (iii) high-density phase (red) and (iv) low-density phase (blue). **b** 3D structural visualization of MNRM illustrating the spatial configuration with a 3:1 volume ratio between low-density (blue) and high-density (red) phases. **c** TEM images of (i) Fe_3_O_4_ NPs and (ii) GO sheets, and (iii) SEM–EDS elemental mapping of the MNRM. **d** UV–Vis–NIR absorption spectra of GO, Fe_3_O_4_ NPs, MRM, and MNRM. **e** Photothermal curves of Fe_3_O_4_ NPs, MRM, and MNRM under 1 sun irradiation with an AM 1.5G filter. **f** Magnetic hysteresis curves of MNRM and MRM. **g** Thermal conductivity of the MNRM, Contrast material I (CNF/PEI/F127) and Contrast material II (CNF/PEI/F127/Fe_3_O_4_ NPs) where the inset is a schematic diagram of GO as a heat transfer bridge in MNRM. The temperature** h** and mass **i** change of MNRM and MNR (containing ~ 44 wt% water) under 1.3 sun light irradiation with/without dynamic magnetic field (~ 80 rpm). **j** Micron-scale reconfiguration motion of the magnetically-driven MNRM inspired by the group behavior of shoals fleeing predators

A simple cross-linking strategy was developed to prepare micro/nanoscale reconfigurable carbon scavenging robots (MNRM) via epichlorohydrin (ECH) cross-linking of a multicomponent system comprising cellulose nano-fibers (CNFs), a temperature-sensitive Pluronic F127 (F127) network, aminated Fe_3_O_4_ nanoparticles (Fe_3_O_4_ NPs), and graphene oxide (GO). The morphology of the MNRM was characterized through nanoscale resolution X-ray computed tomography (nano-CT), transmission electron microscope (TEM), scanning electron microscope (SEM), and energy dispersive spectrometer (EDS) mapping techniques. As shown in Fig. 2a, b, the Fe_3_O_4_ NPs as the high-density phase in MNRM are distributed unevenly. From the SEM–EDS elemental mapping image, it can be observed that the MNRM has an irregular overall shape, with the elements C, N, O, and Fe distributed across its surface (Fig. 2c(iii)). The GO and Fe_3_O_4_ NPs have been demonstrated to effectively convert photon energy from sunlight into thermal energy via non-radiative relaxation processes and carrier diffusion effects upon irradiation. Consequently, the MNRM with both GO and Fe_3_O_4_ NPs integrated exhibits an enhanced solar absorption efficiency of up to 71% (Fig. 2d) and achieves a photothermal temperature of 78 °C under 1 sun irradiation (1000 W m^−2^) (Fig. 2e). Moreover, GO played a dual role by serving as a bridge for transferring heat; thus, the MNRM exhibited enhanced thermal conductivity of 0.24 W/mK, demonstrating a minimum 33.33% increase relative to control materials I (0.18 W mK^−1^) and II (0.17 W mK^−1^)** (**Fig. 2g**)**. This promotes an even temperature rise across the MNRM, despite the photothermal components exhibit non-homogeneous distribution. The reconfiguration properties of the MNRM at the micrometer scale are primarily attributed to the superparamagnetic of the Fe_3_O_4_ NPs (Fig. S4**)**. As illustrated in Fig. 2f, j, the MNRM exhibits strong magnetic properties, enabling an excellent response to external magnetic fields, similar to a school of fish chased by a shark. As shown in Fig. 2h, i, both MNRM and MRM exhibit higher temperatures than the static state under a dynamic magnetic field, yet demonstrate slower rates of weight loss from moisture evaporation. This occurs because under light radiation, the static robot’s surface layer undergoes rapid heating and moisture loss initially. However, due to the lag of heat transfer and uneven exposure to irradiation, the bottom layer was unable to efficiently photothermally convert, thus retaining more moisture. In contrast, the micron-reconfiguration by Fe_3_O_4_ NPs in the dynamic magnetic field has enhanced heat and moisture transfer, effectively prevented localized overheating, and achieved non-contact thermal management.

Additionally, the surface chemical composition of the MNRM was analyzed by X-ray photoelectron spectroscopy (XPS) (Fig. S6). The C 1*s* spectra showed that the MNRM contains 33.90% C-N bonds from PEI at 285.8 eV. Meanwhile, the N 1*s* spectra showed distinct peaks corresponding to the primary (1°) and secondary (2°) and tertiary (3°) amino groups, which are R-NH₂ (23.82%, 398.2 eV), R_2_-NH (38.59%, 397.8 eV), and R_3_-N (37.59%, 398.8 eV). Elemental analysis (EA) was further used to quantify the nitrogen content of MNRM as 23.31%, corresponding to an amino group density of 16.65 mmol g^−1^ (Table S1). In contrast, the temperature-insensitive CO_2_ adsorbent material (MRM), which lacks F127, showed a higher N content of 24.80% and an amino density of 17.71 mmol g^−1^. This suggests that both MNRM and MRM are endowed with abundant amino adsorption sites, giving them the potential to be effective CO_2_ scavenging robots for carbon management in confined spaces.

### Nano-reconfiguration Properties of MNRM

**Fig. 3:**
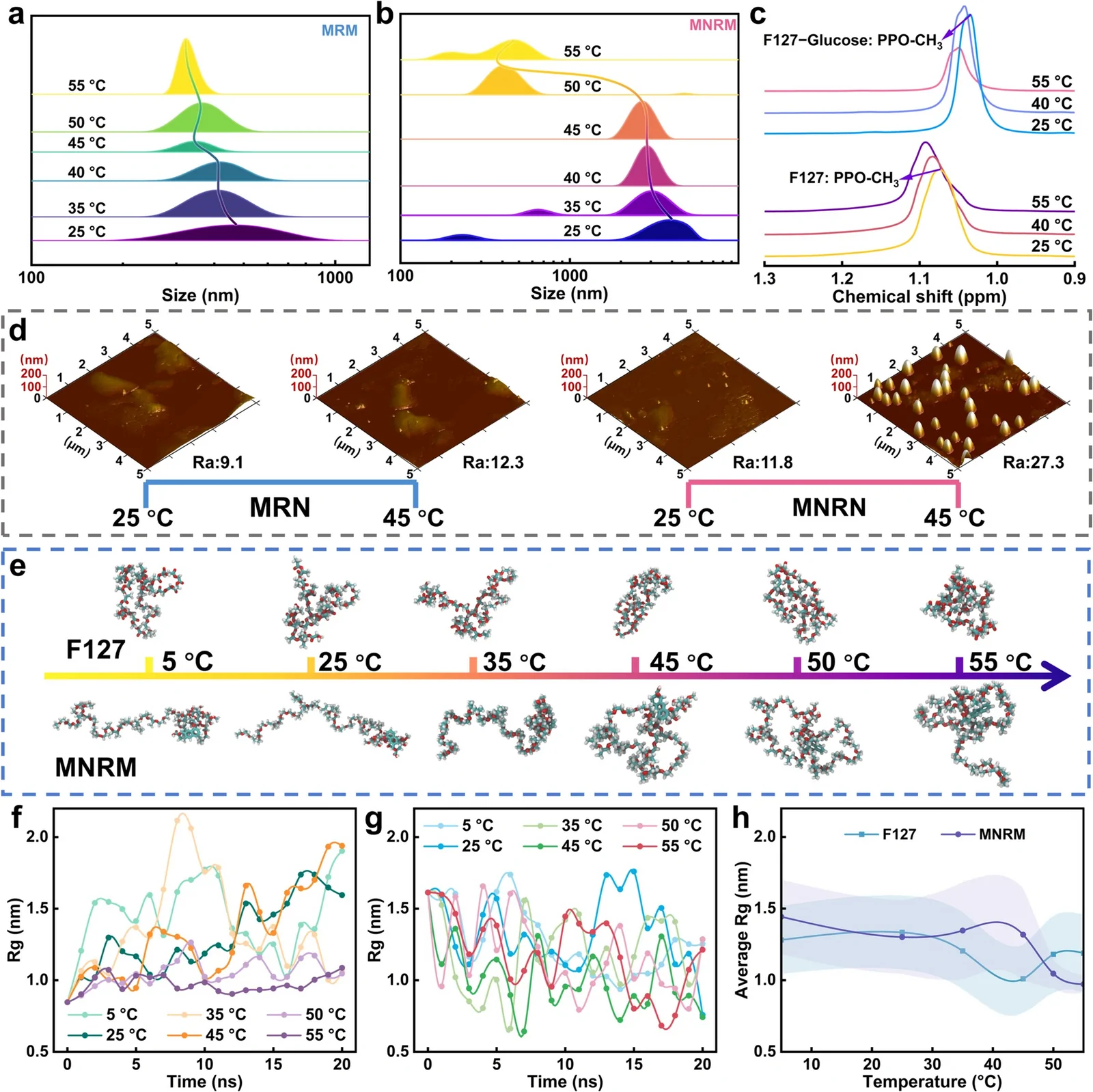
Nano-reconfiguration properties of MNRM. Particle size distribution of **a** MRM and **b** MNRM in water at 25–55 °C. **c** Liquid ^1^H NMR spectra of F127 and CNF-Glucose. **d** AFM imaging of the MRM and MNRM at 25 °C and 45 °C. **e** Atomic trajectory snapshots of F127 and MNRM molecular models at different temperatures. Radius of gyration for **f** F127 and **g** MNRM molecular models at different temperatures over a 0–20 ns period. **h** Average radius of gyration for F127 and MNRM molecular models at different temperatures

The temperature triggered nanoscale reconfiguration of the MNRM was systematically investigated: hydrodynamic size analysis by dynamic light scattering (DLS), surface morphology by atomic force microscope (AFM), bond evolution via variable-temperature XPS and nuclear magnetic resonance spectroscopy (NMR), and molecular dynamics (MD) simulations. DLS revealed a sharp reduction in particle size from > 1000 nm to submicrometric scales at > 45 °C reflecting thermally triggered collapse of the polymeric network (Fig. 3b). AFM results corroborated this transition, showing a 2.3-fold increase in surface roughness (Ra: 11.8 nm at 25 °C to 27.3 nm at 45 °C), indicative of chain reorganization (Fig. 3d). Concomitantly, variable-temperature XPS demonstrated chemical bond redistribution on the surface of MNRM: C–C/C–H content increased from 20.28% to 29.12%, while C–O bonds decreased from 30.42% to 24.50% as temperature rose from 25 to 45 °C (Fig. S7). Notably, the structurally stable MRM exhibited negligible variations in particle size (Fig. 3a), roughness, or bond composition under identical change of temperature, which indicates the critical role of F127 in mediating MNRM’s responsive behavior.

F127 is a temperature-sensitive triblock copolymer composed of poly (ethylene oxide)-b-poly (propylene oxide)-b-poly (ethylene oxide) (PEO-PPO-PEO), where PEO is hydrophilic and PPO is hydrophobic [36]. The thermoresponsive behavior of F127 oligomer model was analyzed through atomic trajectory analysis and radius of gyration (R_g_) measurements across a thermal gradient (25–55 °C). As shown in Fig. 3e, f and h, the molecular movement of F127 exhibited three distinct states as temperature increased. Initially, from 5 to 25 °C, the polymer chains exhibited gradual extension, evidenced by an increased R_g_. As the temperature continued to rise to 45 °C, intermolecular hydrogen bonds strengthened, resulting in increased intermolecular interactions and a curled molecular chain, leading to the smallest average R_g_ at 45 °C. Beyond 45 °C, as the temperature reached 55 °C, the intensified molecular motion caused the breakage of some hydrogen bonds, weakening intermolecular interactions, and the molecular chain re-stretched, slightly increasing the average *R*_g_, but it remained smaller than at the starting point. To impart temperature responsiveness to the MNRM, F127 was cross-linked with CNF, forming a temperature-sensitive CNF-F127 fiber backbone. Unexpectedly, strikingly, CNF-F127 displayed a different molecular chain dynamics compared to pure F127. At 5 °C, the molecular chain of CNF-F127 was most stretched, but it slightly reduced as the temperature increased to 25 °C. Between 25 and 45 °C, the average *R*_g_ increased marginally but not significantly. However, at 50 °C, the average *R*_g_ decreased sharply and continued to decline as the temperature rose (Fig. 3g, h). The results of variable-temperature NMR also illustrate the high sensitivity of the methyl protons of the hydrophobic PPO chain segments of F127 and F127-Glucose to changes in the temperature environment. It is noteworthy that at the equivalent temperature, the displacement amplitude of F127-Glucose is slightly weakened compared to pure F127 and the signal intensity is significantly reduced at 55 °C, suggesting that glucose grafting introduces spatial site resistance and polarity modification to the structure of the copolymer (Fig. 3c). This suggests that the transition temperature of CNF-F127 is influenced by its molecular structure and the interaction between hydrophilic and hydrophobic groups, meaning that the temperature responsiveness of MNRM can be adjusted by altering the F127-to-CNF ratio. Additionally, the nano-reconfiguration induced by the movement of temperature-sensitive molecular chains leads to changes in the amino microenvironment within the MNRM. This plays an important positive role in the ultralow temperature regeneration of MNRM, as described in after section.

### CO_2_ Adsorption Performance of MNRM at Wet and Dry State

**Fig. 4:**
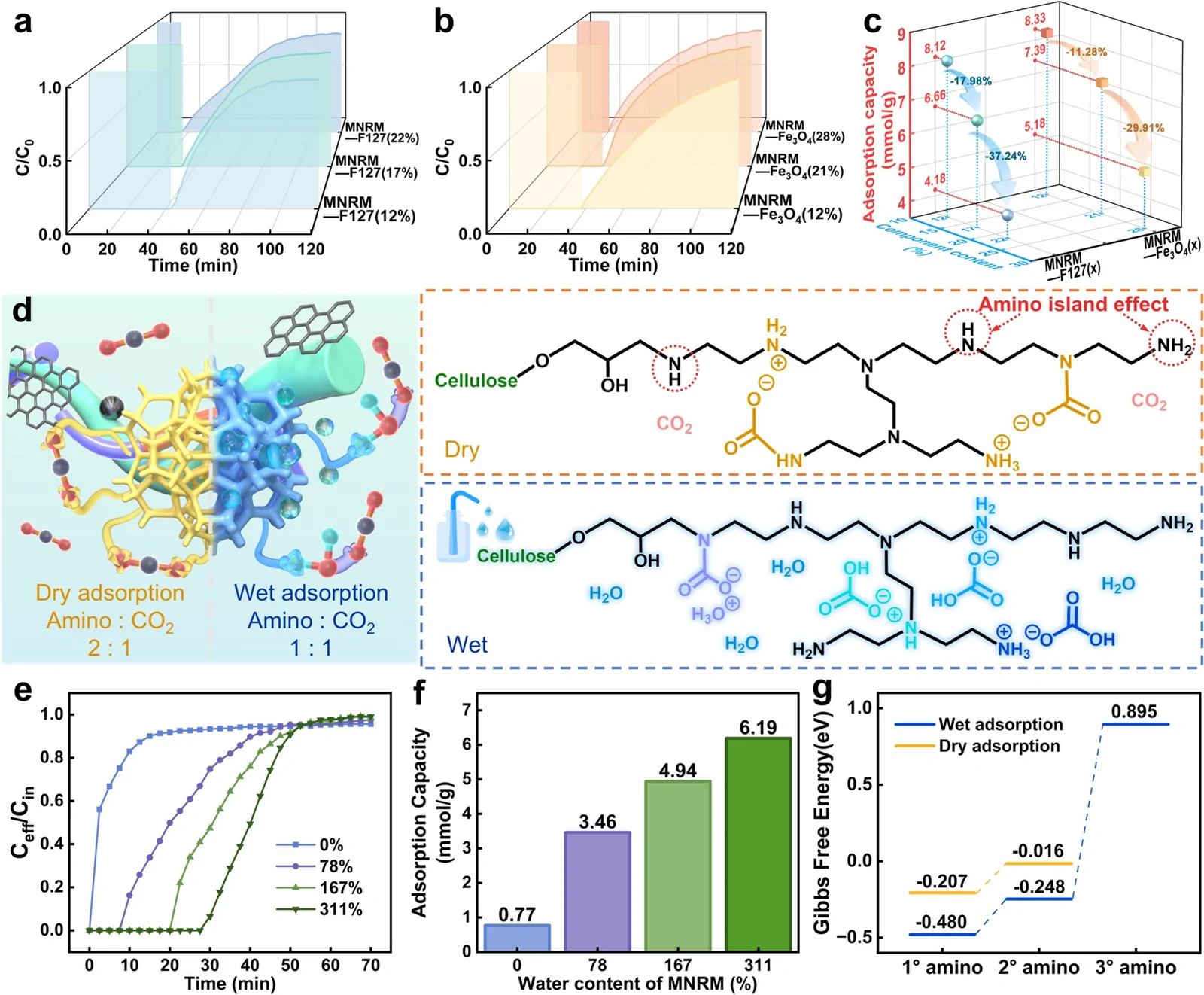
CO_2_ adsorption performance of MNRM at wet and dry state **a** Breakthrough curves for CO_2_ adsorption of MNRM with different F127 contents. **b** Breakthrough curves for MNRM with different Fe_3_O_4_ contents. **c** Corresponding CO_2_ adsorption capacity of MNRM with different F127 and Fe_3_O_4_ contents. **d** CO_2_ adsorption mechanism of MNRM in dry and wet states. **e** Breakthrough curves for CO_2_ adsorption and **f** corresponding adsorption capacities of MNRM at different swelling ratio. **g** Gibbs free energy values of adsorption products of 1°, 2°, and 3°amino in the dry and wet states

Notably, the content of functional modifiers integrated in MNRM inevitably affects its CO_2_ adsorption capacity, as the content of F127 governs the thermal switching behavior of MNRMs, while the loading of Fe_3_O_4_ NPs dictates its photothermal/magnetic responsiveness. To address this critical trade-off, the MNRM-F127 (x) composites with varying F127 contents (12%, 17%, 22%) (Fig. 4a) and MNRM–Fe_3_O_4_ (x) composites with distinct Fe_3_O_4_ NPs loadings (12%, 21%, 28%) (Fig. 4b) were prepared. The results reveal a gradual decline in CO₂ adsorption capacity with increasing F127 or Fe_3_O_4_ NPs content (Fig. 4c). Through systematic optimization of these competing factors, the composite containing 20 % F127 and 15 % Fe_3_O_4_ NPs (denoted as MNRM throughout this work) exhibited excellent CO₂ capture capability, but also maintains a balance of thermal response, photothermal conversion, and magnetic drive characteristic performance. This optimized formulation establishes an essential material platform for developing multifunctional adsorbents in energy-efficient carbon capture systems.

The CO_2_ adsorption properties of the MNRM in both dry and wet states were thoroughly investigated through comparative experiments. As illustrated in Fig. 4e, f the MNRM exhibited a low adsorption capacity of only 0.77 mmol g^−1^ in the dry state, despite its high amino density of 16.65 mmol g^−1^. Remarkably, the CO_2_ adsorption capacity of MNRM exhibits a sharp escalation with hydration level, achieving a maximum of 6.19 mmol g^−1^ when fully swollen at 311% water uptake. The stark disparity between dry and wet adsorption capacities is due to the chemical reaction mechanism and amino groups distribution. In dry conditions, 1° or 2° amino group reacts with CO_2_ in a 2:1 ratio to form carbamate, while 3° amino group does not participate. Of the 16.6 mmol g^−1^ of alkylamino groups in MNRM, only 10.39 mmol g^−1^ of 1° and 2° amino groups are involved in this reaction. Moreover, the competitive occupation of active sites by other molecules or the spatial separation of two cooperating amino groups results in a unique “amino island effect,” which compromises the overall efficiency of their utilization (Fig. 4d). Density functional theory (DFT) calculations showed that the free energy for carbamate formation is –0.207 eV for 1° amino group and –0.016 eV for 2° amino group (Fig. 4g). In contrast, with the presence of water, 1°, 2°, and 3° amino group, each reacts with CO_2_ in a 1:1 ratio, significantly increasing amino groups utilization. The free energies of the ammonium bicarbonate-like products were –0.480, –0.248, and 0.895 eV for 1°, 2°, and 3° amino group, respectively, indicating that CO_2_ adsorption is more favorable in the wet state than in anhydrous adsorption. In particular, the swelling rate of MNRM is as high as 311%, which enhances CO_2_ diffusion and mass transfer efficiency, while also exposing more reactive amino groups to improve adsorption capacity.

### Low-Temperature Regeneration Performance of MNRM Influenced by Nano-reconstruction.

**Fig. 5:**
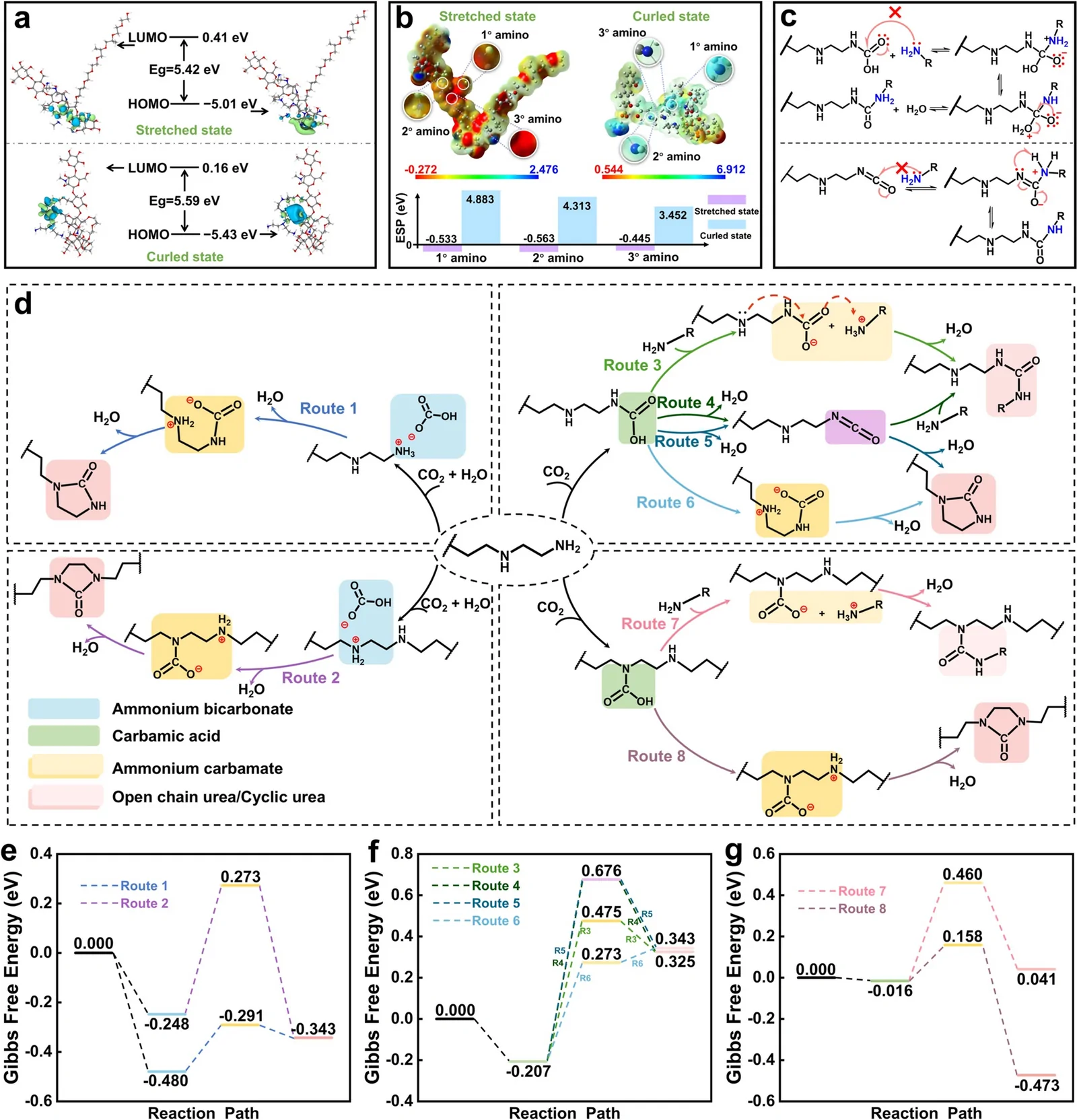
Low-temperature regeneration performance influenced by nano-reconstruction. **a** The highest occupied molecular orbital (HOMO), lowest unoccupied molecular orbital (LUMO) and **b** amino surface electrostatic potential of MNRM at stretched and curled state. **c** Process of nucleophilic attack and electron rearrangement by the amino group on carbamic acid/isocyanate. **d** Formation pathways of urea and **e–g** the corresponding Gibbs free energy profiles

The high regeneration temperature of amino-containing carbon scavenging materials is often related to the formation of urea structures. Given that 62.41% of the amino groups in the MNRM are 1° and 2° aminos, which react readily with CO_2_, DFT simulations were used to examine the structural changes of these two groups during desorption. As illustrated in Fig. 5d, Route 1 and Route 2 show the formation of cyclic urea after dehydration of ammonium bicarbonate, the reaction product of 1° and 2° aminos with CO_2_ and H_2_O, respectively. Although 1° aminos are more likely to take up CO_2_ than 2° aminos, they are also more likely to be dehydrated to produce urea, as shown in the Gibbs free energy values in Fig. 5e. When carbamic acid serves as the CO₂ adsorption product, two distinct pathways emerge [37, 38]: (1) Carbamic acid is affected by neighboring amino groups to form ammonium carbamate, which is then dehydrated to form urea (including Routes 3, 6, 7, and 8); or (2) the carbamic acid dehydrates to form isocyanate before evolving to urea (Routes 4 and 5). Both pathways can result in open-chain urea between molecular chains or cyclic urea within chains. Notably, the second pathway cannot occur in 2° aminos due to steric restrictions, since the presence of two alkyl groups prevents the formation of isocyanate structures of this route. The free energy values of these routes illustrate that the formation of isocyanate-like structures from carbamic acid requires higher energy (0.676 eV for Route 4 and Route 5) compared to ammonium carbamate formation (0.475 eV for Route 3 and 0.273 eV for Route 6). It is noteworthy that MNRM molecular chains undergo nano-reconfiguration during thermal regeneration. The highest occupied molecular orbital (HOMO), the lowest unoccupied molecular orbital (LUMO) and the amino surface electrostatic potential of the representative MNRM structures that stretch at low temperatures and curl up at high temperatures were calculated using DFT analysis.

The results show that the amino groups are primarily located on the HOMO orbitals. As the molecule structure contracts at higher temperatures, the HOMO energy level decreases from − 5.01 to − 5.43 eV as the structure of the molecule changes from stretching to curling (Fig. 5a). Moreover, amino surface electrostatic potential of MNRM increased significantly from − 0.533 (1° amino), − 0.563 (2° amino), and − 0.445 eV (3° amino) at stretched state to 4.883, 4.313, and 3.452 eV at curled state, respectively (Fig. 5b). These changes result in the amino groups acting as the nucleophilic reagent that initiate the nucleophilic attack on the carbamic acid or isocyanate is blunted, with reduced nucleophilicity and electron rearrangement, making it difficult to carry out the reaction that generates the difficult-to-decompose urea structure (Fig. 5c). As a result, the nano-reconfiguration triggered by temperature stimulation is the key to the low-temperature regeneration performance of the MNRM.

### Energy-efficient Regeneration of MNRM

**Fig. 6:**
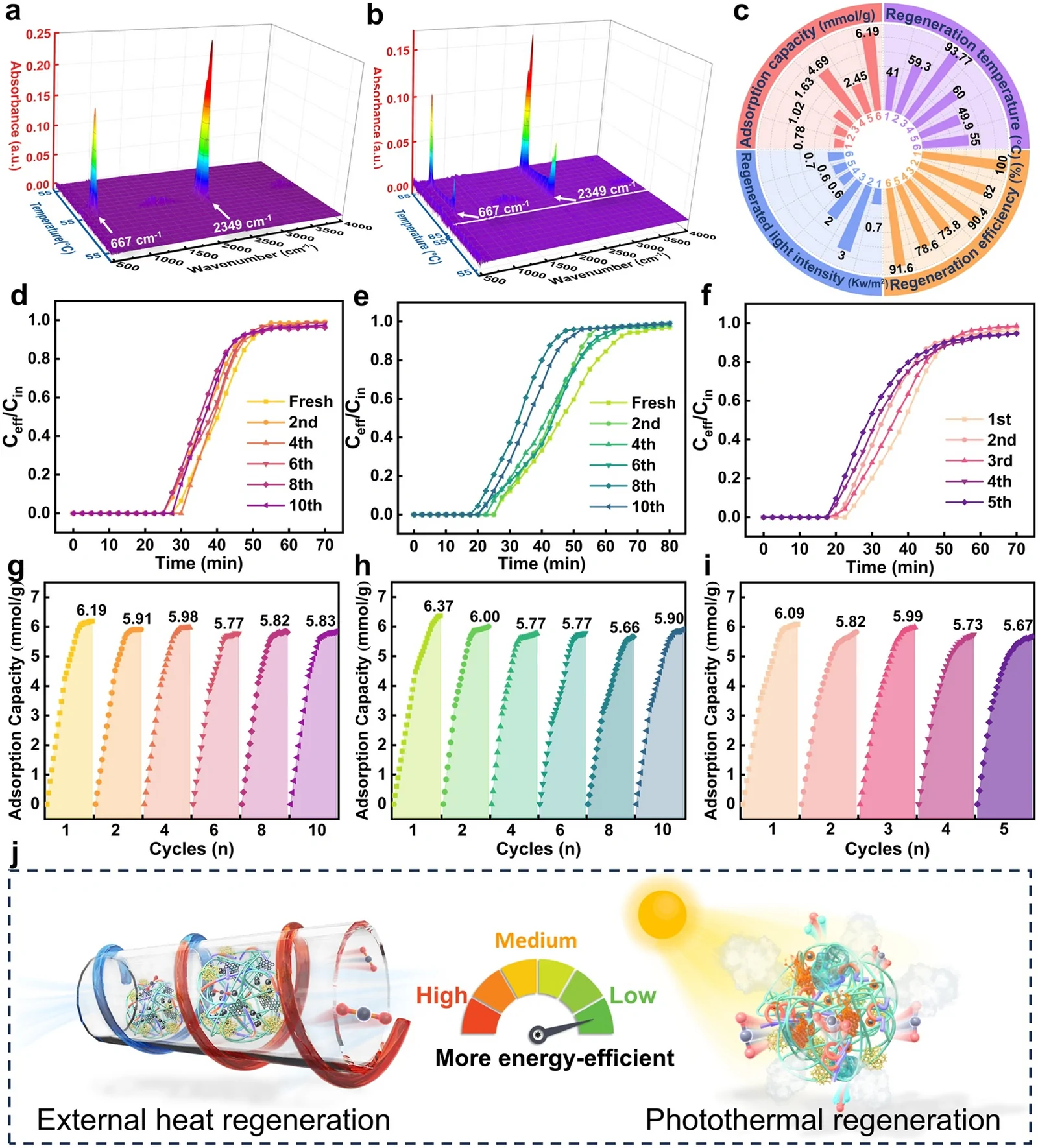
Energy-efficient regeneration of MNRM. **a** 3D TG-IR spectra of MNRM at 55 °C. **b** 3D TG-IR spectra of MRM at 55 °C and 85 °C. **c** Comprehensive comparison of MNRM with other photo-triggered regeneration CO_2_ adsorption materials^*^ in four dimensions: adsorption capacity, photo regeneration temperature, photo regeneration efficiency, and photo intensity. **d** Breakthrough curves for CO_2_ adsorption and **g** the corresponding adsorption capacities of MNRM (cyclic regeneration condition: external hydrothermal heating at 55 °C). **e** Breakthrough curves for CO_2_ adsorption and **h** the corresponding adsorption capacities of MRM (cyclic regeneration condition: external hydrothermal heating at 85 °C). **f** Breakthrough curves for CO_2_ adsorption and **i** the corresponding CO_2_ adsorption capacities of MNRM (cyclic regeneration condition: photothermal heating at 55 °C). **j** Schematic of MNRM's dual regeneration mode (external hydrothermal and photothermal regeneration). *^1^CBP: A CO_2_ breathing paper by coupling imidazole (EMI) with carbon nanotube (CNT) [39]. ^2^PEI/FS/CB: A photothermal adsorbent composite of polyethyleneimine (PEI), fumed silica (FS), and carbon black (CB, 10 wt%) with a dry adsorption capacity of 45 mg g^−1^ (converted to 1.02 mmol g^−1^), which can be heated up to 59.3 °C under 3 kw m^−2^ radiation [24]. ^3^aminosilia@MXene (7 wt%): A composite material composed of fumed silica as a framework, MXene as a photothermal conversion component, and (3-aminopropyl) triethoxysilane (APTES) as a CO₂ adsorption site [40]. ^4^SP-3-700: Biomass porous carbon prepared by high-temperature calcination followed by KOH activation using succulent plants as precursors, with a static adsorption capacity of 4.19 mmol g^−1^ at 25 °C [41]. ^5^Thermomorphic solvents containing carboxyl-functionalized multiwalled carbon nanotubes (MWCNT-COOH) with CO_2_ absorption capacity of 0.1081 g g^−1^ (converted to 2.45 mmol g^−1^) and regenerated at ~ 325 K (converted to 49.9 °C) [42]. ^6^MNRM: This work

The nano-reconfigurable properties of the MNRM allow it to achieve exceptionally low regeneration temperatures. As demonstrated by the 3D thermogravimetric analysis-infrared spectrometry (TG-IR) spectra of the MNRM, a distinct absorption peak between 2200 and 2500 cm^−1^, corresponding to the asymmetric stretching vibration of CO_2_, was observed when the temperature was held at 55 °C (Fig. 6a). This indicates that the MNRM can successfully desorb CO_2_ at this temperature. Regeneration experiments confirmed that after 10 regeneration cycles, the MNRM maintained a high adsorption capacity of 5.83 mmol g^−1^, with a regeneration rate of 94.18% (Fig. 6d, g). In addition, the regeneration energy consumption of MNRM was estimated to be approximately 314.86 J g^−1^ by differential scanning calorimetry (Fig. S21). Furthermore, the long-term cyclic temperature stability and durability of MNRM under complex conditions were further verified (Figs. S11 and S20). These results demonstrate the excellent low-temperature regeneration performance of the MNRM and its potential for long-term use in adsorption–desorption cycles.

The CO_2_ adsorption and cyclic regeneration of MRM without F127 were further tested to determine whether the ultralow-temperature regeneration properties of MNRM was attributable to the temperature sensitivity and nano-reconstructive properties imparted by F127. The 3D TG-IR spectra of MRM showed no substantial CO_2_ absorption peak at 55 °C. It was only when the temperature was elevated to 85 °C that a characteristic peak for CO₂ appeared near 2349 cm^−1^ (Fig. 6b). Additionally, when MRM was regenerated at a lower temperature (55 °C), its CO_2_ adsorption capacity dropped significantly to 4.09 mmol g^−1^ after 10 cycles, suggesting incomplete regeneration (Fig. S14). Full regeneration was only achieved at 85 °C, where MRM demonstrated a regeneration rate of 92.69% after 10 cycles (Fig. 6e, h). This indicates that MRM does not possess the same low-temperature regeneration properties as MNRM. These findings unequivocally confirm that the ultralow-temperature regeneration capability of the MNRM is the result of temperature-sensitive, stimulus-responsive nano-remodeling imparted by F127.

To further minimize the regenerative energy consumption of the carbon scavenging process, inexhaustible solar energy was employed for the photo-triggered regeneration of the MNRM. Under 1 sun of radiation (1000 W m^−2^), the temperature of the MNRM can reach 78 °C, which exceeds its required regeneration temperature. Therefore, only 0.7 sun (700 W m^−2^) of radiant power was needed in the photothermal regeneration experiments to heat the MNRM to 55 °C. As shown in Fig. 6f, i, the MNRM was successfully regenerated 10 times under 0.7 sun of simulated sunlight, achieving a regeneration rate of 91.6%. This result highlights that the ultralow regeneration temperature of 55 °C for the MNRM allows for efficient photothermal regeneration using only low-power-density light. Even when exposed to unadjusted high-energy light, the magnetically-driven microscopic reconstruction characteristics of the MNRM help prevent thermal damage from localized high temperatures. Compared to recently reported carbon capture materials with photo-triggered regeneration, MNRM demonstrates an outstanding optimal four dimensions: balance of high CO_2_ adsorption capacity and minimal regeneration temperature. A comparative analysis of recently reported carbon capture materials with light-triggered regeneration reveals that MNRM exhibits the overall performance in four key metrics: a high CO_2_ adsorption capacity of 6.19 mmol g^−1^, a low photothermal regeneration temperature of 55 °C, 91.6% photothermal regeneration efficiency, and a regeneration light irradiation intensity of 0.7 sun (Fig. 6c). This multidimensional optimization endows MNRM with exceptional energy efficiency, enabling its deployment across diverse CO_2_ capture scenarios, notably in closed-loop life-support systems where low-energy regeneration and operational sustainability are critical.

### CO_2_ Management Capability of MNRM for Life-Support Systems

**Fig. 7:**
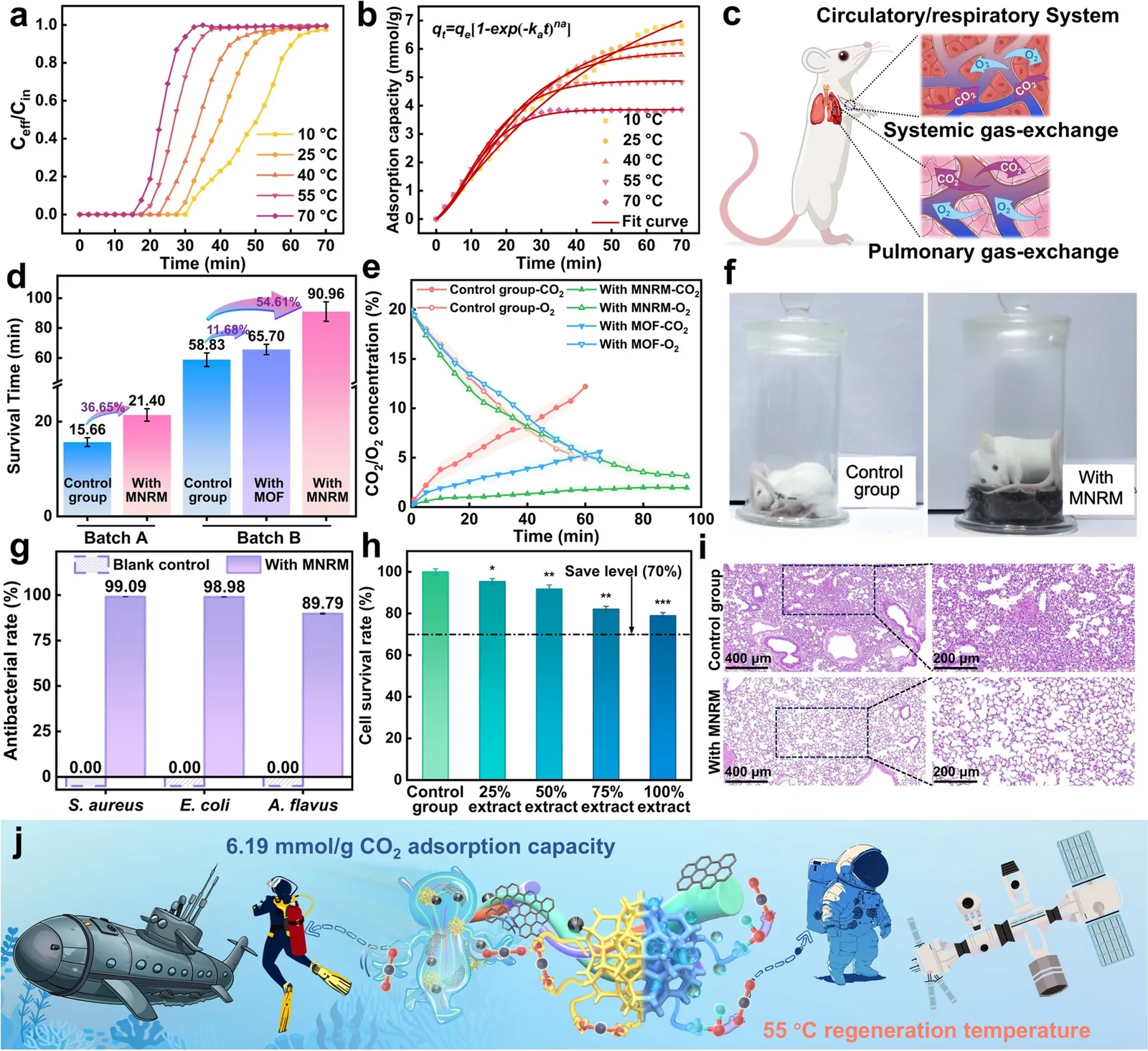
CO_2_ management capability of MNRM for life-support systems. **a** Breakthrough curves of MNRM at different temperatures and **b** the corresponding adsorption capacities were fitted by the Avrami model. **c** Circulatory/respiratory system of mice. **d** Average survival time for mice of batches A and B (n = 3 mice per group). **e** Real-time O_2_ and CO_2_ average concentration curves in sealed containers of mice. **f** Optical photograph of device for confined-space survival experiment of batch A mice. **g** Antimicrobial rate of MNRM against *Staphylococcus aureus* (*S. aureus*), *Escherichia coli* (*E. coli*), and *Aspergillus flavus* (*A. flavus*) (n = 3 per group). **h** Viability of BEAS-2B cells. (n = 3; *P < 0.05, **P < 0.01, and ***P < 0.001) **i** H&E staining of lung sections from asphyxiated mice.** j** Schematic diagram of MNRM for carbon management in the typical confined space

The process of the CO_2_ and amino group reaction is governed by a complex interplay between thermodynamics and kinetics. The effect of different temperatures on the adsorption behavior and kinetic analysis of MNRM was explored (Fig. 7a, b). The results indicate that the adsorption capacities of the MNRM decrease as temperature increases within the studied range. Specifically, at a low temperature of 10 °C, the MNRM achieved peak adsorption capacities of 7.14 mmol g^−1^. In contrast, when the temperature was increased to 70 °C, their adsorption capacities dropped dramatically to 3.8 mmol g^−1^. This suggests that the inhibition of the exothermic reaction by high temperatures dominated the adsorption process. To further investigate the adsorption behavior and kinetics, dynamic data fitting based on different temperatures revealed that the Avrami kinetic model (R^2^ = 0.9961–0.9983) is more applicable compared to the pseudo-primary and pseudo-secondary models, indicating that CO₂ adsorption is not a single physical or chemical process but a synergistic effect of both (Table S2). Further analysis of the mass transfer model revealed that the membrane diffusion process is the main controlling step of the adsorption rate (Fig. S17). However, the high swelling characteristics of the material can reduce the mass transfer resistance at the initial stage, thus enhancing the adsorption rate and capacity.

Given the unique characteristics of confined-space environments, there is a pressing need for efficient, low-energy CO₂ removal materials. Breakthrough curve analysis of MNRM reveals complete CO₂ capture within the initial 27.5 min phase of dynamic adsorption testing at 25 °C, accompanied by an adsorption half-life of 20.5 min (Figs. 7a and S18). This suggests that it has great potential as a key decarbonization component for life-support systems. To further validate its capability, an experiment was conducted simulating survival in an extremely confined environment, where two batches of mice were exposed to both hypoxia and the gradual accumulation of highly concentrated CO_2_. As illustrated in Video S2, the batch A mice initially displayed high activity levels and increased respiration rates upon entry into the sealed canisters, indicating an immediate physiological stress response to the environmental changes. However, as oxygen was depleted and CO_2_ levels rose, the vitality of the mice continued to decline, marked by slowed movements, weakness, and eventual paralysis. Compared to the control group, the mice in the experimental group exhibited a notable delay of approximately 4 ~ 5 min in the exhaustion period, likely because of the timely removal of CO₂ (Fig. S24). The results of gas monitoring in a closed environment for mice in B batch showed that the O_2_ consumption rates of mice in each group did not differ significantly, and the O_2_ concentration at the time of death was approximately 4% in all groups. However, there were significant differences in the cumulative CO_2_ concentrations at the time of death among the groups. The blank control group reached as high as 12.2%, followed by the commercial MOF (ZIF-8) reference control group at 5.6%, while the MNRM experimental group had the lowest concentration at just 1.95%. These physiological environmental data indicate that MNRM possesses highly efficient real-time removal capabilities for CO_2_ generated in closed environments. Following the asphyxiation experiment, murine lung tissues underwent hematoxylin–eosin (H&E) staining for histopathological evaluation, which revealed distinct intergroup pathological profiles (Fig. 7i). According to the five-grade classification of tissue lesions, the control group specimens exhibited grade 3 lesions, characterized by widespread alveolar hemorrhage, interstitial edema, and alveolar septal disruption resulting in structural deformation. This damage is likely owing to impaired gas exchange, resulting in lower oxygen partial pressure, elevated CO_2_ partial pressure, and a disturbed acid–base balance in the blood. In contrast, the lungs of experimental group with MNRM maintained structural integrity (grade 1), showing only mild interstitial edema and intra-alveolar material exudation. Therefore, despite differences in baseline survival time between the two batches of mice that may be attributed to differences in age and body weight, MNRM significantly extended survival time in both batch A (36.65%) and batch B (54.61%) mice. This cross-batch consistency indicates that MNRM can produce significant survival benefits in mouse models with different physiological characteristics. These results highlight the exceptional CO_2_ scavenging ability of the MNRM in confined spaces, effectively overcoming the carbon asphyxiation challenge demonstrated in the mouse model. This underscores the potential of the MNRM as a critical component for carbon management in life-support systems within confined spaces such as submarines and space stations, and its promising application in emergency rescue scenarios (Fig. 7j). By rapidly adsorbing and reducing CO_2_ concentrations, the MNRM can extend the “golden rescue window” for trapped people, increasing survival probabilities and the success rate of rescue operations.

Considering the MNRM as a potential candidate for carbon scavenging technology in life-support systems, it is crucial that it meets high standards of antimicrobial performance during both storage and use. To evaluate the broad-spectrum antimicrobial efficacy of the MNRM, three representative microorganisms were selected: *S. aureus* (gram-positive bacteria), *E. coli* (gram-negative bacteria), and *A. flavus* (fungal). As illustrated in Figs. 7g and S28, the MNRM demonstrated significant antimicrobial activity against all three microorganisms compared to the untreated control group. The antimicrobial rates against *E. coli*, *S. aureus*, and *A. flavus* were 99.08%, 98.97%, and 89.79%, respectively. These results indicate that the MNRM possesses strong antimicrobial properties in complex and diverse microbial environments, enhancing the overall hygiene of life-support systems and extending their safe operational life. Further assessment of the biocompatibility of MNRM was conducted through in vitro cytotoxicity experiments. Human normal lung epithelial cells (BEAS-2B cells) were used, exposed to MNRM extract solutions at different concentrations. The results showed (Fig. 7h) that the cell survival rate in the group administered the 100% concentration extract was 78.99%, which was significantly higher than the 70% biological safety threshold. In the 25% extract group, the cell survival rate was as high as 97.51%. These data fully prove the excellent biocompatibility of MNRM.

## Conclusions

In this study, we developed micro- and nanoscale reconfigurable robots MNRM designed for confined spaces with strict volume and energy constraints, enabling energy-efficient carbon removal. Inspired by the behavior of fish schools, this robot is composed of cellulose nano-fibers, PEI, F127, Fe_3_O_4_ NPs, and GO using a simple cross-linking strategy. The resulting MNRM exhibits a high density of amino groups, along with excellent thermosensitivity, photothermal conversion, and magnetic actuation properties. Because of the high density of amino groups and favorable swelling characteristics, the MNRM demonstrated rapid and efficient CO_2_ removal at low concentrations, achieving an adsorption capacity of 6.19 mmol g^−1^. Additionally, the temperature-sensitive nano-reconfiguration of the MNRM leads to a reduction in the HOMO energy level and an increase in the electrostatic potential on the amino surface. This reduces the nucleophilic reactivity of the amino group, effectively preventing the generation of difficult-to-break-down urea structures. Consequently, the MNRM exhibits an ultralow regeneration temperature of 55 °C, the lowest reported for amino-based adsorbents, and maintains efficient performance through ten regeneration cycles at this temperature. Furthermore, the exceptional photothermal conversion capability of the MNRM enables effective utilization of solar energy for light-triggered regeneration. Its magnetically-driven properties prevent localized overheating caused by prolonged high-energy light exposure, improving photothermal efficiency and reducing overall energy consumption during carbon scavenging. In practical applications, the MNRM efficiently removes low concentrations of CO_2_, extending the survival time of two batches mice in confined spaces by 36.65% and 54.61%, respectively. This study makes a significant contribution to the development of energy-efficient carbon removal materials for specialized environments in confined spaces, as well as for broader carbon capture applications.

## Supplementary Information

Below is the link to the electronic supplementary material.

Supplementary file 1 (DOCX 7082 KB)

Supplemental Video 1 (MP4 431 KB)

Supplemental Video 2 (MP4 2845 KB)

Supplemental Video 3 (MP4 6694 KB)
