# Supplementary material for: Micro/Nano-Reconfigurable Robots for Intelligent Carbon Management in Confined-Space Life-Support Systems
Source: Nanomicro Lett. 2025 Nov 4;18:80. doi: 10.1007/s40820-025-01932-9 (PMC12583435; doi:10.1007/s40820-025-01932-9)
Supplement: Supplementary file 1 — (DOCX 7082 KB) [file 40820_2025_1932_MOESM1_ESM.docx]

Supporting Information for

**Micro/Nano-Reconfigurable Robots for Intelligent Carbon Management in Confined Space Life-Support Systems**

Wei Lu^1^, Rimei Chen^1^, Lianlong Zhan^1^, Qin Xiang^1^, Renting Huang^1^, Lei Wang^1^, Shuangfei Wang^1^, and Hui He^1,^*

^1^ Guangxi Key Laboratory of Clean Pulp & Papermaking and Pollution Control, School of Light Industry and Food Engineering, Guangxi University, Nanning 530004, P. R. China

*Corresponding author. E-mail: [gxuhh@gxu.edu.cn](mailto:gxuhh@gxu.edu.cn) (Hui He)

**S1 Experimental Section**

**S1.1 Preparation of Carboxylated Cellulose Nanofibers (CNF)**

First, 5.00 g of CF was uniformly dispersed in 300.0 mL of distilled water, followed by the addition of 0.06 g (0.4 mmol) TEMPO, 0.30 g (2.0 mmol) NaClO and 4.52 g NaClO_2_. After stirred at 60 °C for 16 h, the mixture was added 10.0 mL anhydrous ethanol to end the reaction. The resulting product was washed with distilled water and diluted to 0.6 wt%, then homogenized at high pressure to obtain uniform suspension. The obtained carboxylated cellulose nanofibers suspension was labelled as CNF.

**S1.2 Preparation of Microscale Reconfigurable Robots (MRM) and Other Contrasting Materials**

The MRM was prepared using the same method and feed ratio as MNRM, but without adding F127. For thermal conductivity comparisons, Contrast Material I (CNF/PEI/F127) and Contrast Material II (CNF/PEI/F127/Fe_3_O_4_ NPs) were cross - linked using the same process.

**S1.3 Materials Characterization**

Scanning electron microscopy (SEM) (ZEISS SIGMA500, Germany, gold spraying of sample), transmission electron microscopy (TEM) (FEI Talos F200S), atomic force microscopy (AFM) (Hitachi 5100N, Japan, Test mode: Tapping mode) and nanoscale resolution X-ray computed tomography (nano-CT) (Bruker skysCan2211, Germany) were employed to characterize the morphology of the samples. In situ variable temperature X-ray photoelectron spectroscopy (PHI5000 Versaprobe III XPS), ^1^H NMR (Bruker AV 500M), and elemental analysis (EA) (Elemantar Vario EL cube, Germany) were employed to investigate the physicochemical properties of the samples. UV/Vis/NIR diffuse reflectance testing: integrating sphere absorption mode, lambda 1050. Vibrating sample magnetometers (VSM) hysteresis loop test: LakeShore7404, magnetic field slow sweep: ±2T. Hot Disk thermal constant analyzer: TPS2500S, Sweden. Thermogravimetric infrared (TG-IR): PerkinElmer TGA4000+SP2.

The hydrodynamic diameter and surface potential measurements (Malvern Zetasizer Nano ZS90) were performed using the dynamic light scattering (DLS) technique.

**S1.4 Swelling Degree Test**

$$\begin{aligned} S=\frac{\left( m_{2}-m_{1} \right)}{m_{1}}\times100\% \#\left（ S1 \right） \end{aligned}$$

where *S* is the swelling degree (%), *m_1_* is the weight of the dried adsorbent (g), *m_2_* is the weight of adsorbent (g) after reaching swelling equilibrium in water, and the swelling time was 1 h.

**S1.5 Amino Content Calculation**

$$\begin{aligned} \rho=\frac{m\cdot W_{t}}{m\cdot M}\cdot1000 \#\left（ S2 \right） \end{aligned}$$

where $\rho$ is the amino density (mmol/g), *m* is the weight of sample used in EA (g), $W_{t}$ is the element proportion (wt %), and *M* is the molar mass (g/mol).

**S1.6 CO_2_ Adsorption Procedure**

The CO_2_ regeneration procedure is similar to that previously reported in the literature [S1]. Specifically, 0.55 g of the carbon scavenging material was pre-soaked in distilled water for 1 h. Afterward, the material was filtered and packed into a column (Φ = 1.3 cm). A gas mixture of CO_2_ / N_2_ (with CO_2_ comprising 5% by volume) was introduced at a flow rate of 35 mL/min, and the concentration of CO_2_ at the outlet of the adsorption column was measured using a gas chromatograph (Techcomp GC7980). Breakthrough curves were used to assess the CO_2_ adsorption performance of the samples.

The adsorption capacity (Q) was calculated as follows:

$$\begin{aligned} Q={\int_{0}^{t} \left( C_{in}-C_{eff} \right)V dt}/{(Vm}\cdot W) \#\left（ S3 \right） \end{aligned}$$

where *Q* is the adsorption capacity (mmol/g), *t* refers to the adsorption time (min), and *C_in_* and *C_eff_* represent the influent and effluent concentrations of CO_2_ (vol%), respectively. *V* represents the total flow rate, 35 mL/min. *Vm* is the molar volume of gas, e.g. 24.5 mL/mmol at 25 °C and 1 atmosphere. *W* refers to the weight of the sample (g).

**S1.7 Regeneration Procedure**

External hydrothermal regeneration: The recirculating hot water with a preset temperature (85 or 55 °C) was introduced into the jacket of the adsorption column. And N_2_ gas was flowed at 80 mL min^-1^ through the column to regenerate the carbon scavenging material.

Photothermal regeneration: The material was photothermally desorbed using a solar simulator (CEL-S500/350, Aulight, China) equipped with an AM 1.5 filter. The adsorbent temperature was monitored using an infrared temperature-sensing camera (Teledyne FLIR E85).

The regenerated material was reintroduced into the CO₂ adsorption procedure, and its regeneration performance was evaluated by the secondary CO_2_ adsorption capacity.

$$\begin{aligned} \gamma=\frac{Q_{n}}{Q_{1}}\times100\% \#\left( S4 \right) \end{aligned}$$

where $\gamma$ is the regeneration rate (%). *Q_n_* is the adsorption capacity of the regenerated material and *Q_1_* is the initial adsorption capacity of the material.

**S1.8 Molecular Dynamics Simulations**

All-atom molecular dynamics simulations were used to investigate the structural and dynamic changes of polymer F121 and the temperature-sensitive fiber backbone (CNF-F127) at different temperatures. PEO_15_-PPO_10_-PEO_15_ and (C_6_H_10_O_5_)_3_-PEO_15_-PPO_10_-PEO_15_ were constructed as representative models of F127 and CNF-F127, respectively. Prior to molecular dynamics (MD) simulations, the system is energy-minimized via the steepest descent method to reduce initial high local energies from atomic overlaps. Then, a 0.05 ns MD simulation is run under NVT conditions, followed by 200.0 ns of MD equilibration under NPT conditions. The temperature is maintained at 300.0 K with the Berendsen thermostat, and the pressure at 1.0 bar semi-isotopically via the Parrinello-Rahman barostat. Bond lengths are constrained by LINCS, and long-range electrostatics are managed by PME with a 12.5 Å cutoff. The model involves a polymer chain centered in a 4.0 nm × 4.0 nm × 4.0 nm box, solvated with ~2500 H_2_O molecules. OPLS-AA force field parameters generated via the PolyParGen tool are applied. MD is simulated using GROMACS, and visual analysis is done with VMD [S2, S3].

The atomic trajectory was analyzed using the radius of gyration (Rg).

$$\begin{aligned} R_{g}=\sqrt{\frac{1}{M}\sum_{i} m_{i}{(r}_{i}-r_{cm})^{2}} \#\left( S5 \right) \end{aligned}$$

Where $r_{cm}$ is the center of mass of the group, $M$ is the mass of the group, and the

Sum is over all atoms in the group.

**S1.9 Quantum Chemistry Simulations**

Highest Occupied Molecular Orbital (HOMO), Lowest Unoccupied Molecular Orbital (LUMO) and the molecular electrostatic potential (ESP) were used to investigate the stretched and curled conformations of representative MNRM structures. All studied geometries were optimized via Gaussian 09 D01 package with ultrafine integration grids at Becke's three-parameter hybrid exchange function with Lee–Yang–Parr gradient-corrected correlation functional (B3LYP) including Grimme’s DFT-D3(BJ) empirical dispersion correction and the def2-SVP basis set level of theory [S4]. Moreover, a further harmonic frequency was calculated at the same level to ensure that there are no imaginary frequencies for optimized geometries, i.e. the structures located at the minimum of the potential energy surface. The molecular electrostatic potential (ESP) was yielded from Gaussian 09 and GaussView 5.0.9, in which the molecular surface is defined by the electron density equal to 0.001 a.u. that is suggested by Bader et al. to manifest molecular van der Waal surface. And the minimum and maximum values of ESP were gained from Multiwfn 3.8 [S5].

**S1.10 DFT Calculations**

Conformation search at the GFN2-xTB semiempirical level is employed, followed by DFT calculations, to obtain the optimal structures [S6]. Conformation search was performed by iMTD-GC conformer search workflow implemented in the Crest program [S7]. The total Meta-Dynamics simulation time was 600 ps for each system. The best conformers were optimized at PBE0 /def2-SVP level with Grimme's D3BJ empirical dispersion correction [S8]. All DFT calculations were performed using Gaussian 16 program.

**S1.11 Kinetics of CO_2_ Adsorption**

In order to investigate the adsorption behavior and the adsorption kinetics of MNRM and MRM, the pseudo-first-order, pseudo-second-order and Avrami kinetic model were applied to fit the dynamic CO_2_ adsorption data at different temperatures.

The computational formula of pseudo-first-order kinetic model is shown below:

$$\begin{aligned} q_{t}=q_{e}\left( 1-ⅇ^{-k_{f}t} \right) \#\left( S6 \right) \end{aligned}$$

The computational formula of pseudo-second-order kinetic model is shown below:

$$\begin{aligned} q_{t}=\frac{k_{s}{q_{e}}^{2}t}{1+q_{e}k_{s}t} \#\left( S7 \right) \end{aligned}$$

The computational formula of Avrami kinetic model is shown expressed as below:

$$\begin{aligned} q_{t}=q_{e}\left[ 1-\exp\left( 1-k_{a}t \right)^{n_{a}} \right] \#\left( S8 \right) \end{aligned}$$

where *q_t_* represents the amount of adsorption at a given time point, *q_e_* refers to the amount of adsorption reaches equilibrium, and *n* represents the order of kinetic equation. *k_s_* (g/ (mmol min)), *k_f_* (min^-1^), and *k_a_* (min^-1^) refer to all rate constants. *t* represents the time elapsed since the adsorption process began. Besides, for Avrami model, when 1 < *n_a_* < 2, both chemisorption and physisorption are the rate-controlling factors. When *n_a_* =1 or *n_a_* = 2, the chemisorption and physisorption are the rate-controlling factors in the adsorption process.

**S1.12 Investigation of CO_2_ Diffusion Mechanism**

Boyd’s model, interparticle diffusion model and intraparticle diffusion model are employed to analyse the experimental kinetic data so as to further understand the CO_2_ diffusion mechanism and the rate control step.

The computational formula of interparticle diffusion model is shown below:

$$\begin{aligned} \frac{\partial q}{\partial t}=D_{c}\left( \frac{\partial^{2}q}{\partial r^{2}}+\frac{2}{r}\frac{\partial q}{\partial r} \right) \#\left( S9 \right) \end{aligned}$$

$$\begin{aligned} 1-\frac{q_{t}}{q_{e}}=\frac{6}{\pi^{2}}\exp\left( -n^{2}k_{1}t \right) \#\left( S10 \right) \end{aligned}$$

$$\begin{aligned} k_{1}=\frac{\pi^{2}D_{c}}{r^{2}} \#\left( S11 \right) \end{aligned}$$

The computational formula of intraparticle diffusion model is shown below:

$$\begin{aligned} q_{t}=k_{2}t^{\frac{1}{2}}+b \#\left( S12 \right) \end{aligned}$$

The computational formula of Boyd’s film-diffusion model is shown below:

$$\begin{aligned} F=1-\frac{6}{\pi^{2}}\sum_{n=1}^{\infty} \frac{1}{n^{2}}exp\left( -n^{2}B_{t} \right) \#\left( S13 \right) \end{aligned}$$

$$\begin{aligned} F=\frac{q_{t}}{q_{e}} \#\left( S14 \right) \end{aligned}$$

For *F* > 0.85,

$$\begin{aligned} B_{t}=-0.4977-\ln\left( 1-F \right) \#\left( S15 \right) \end{aligned}$$

And for *F* < 0.85,

$$\begin{aligned} B_{t}=\left( \sqrt{\pi}-\sqrt{\pi-\left( \frac{\pi^{2}F}{3} \right)} \right)^{2} \#\left( S16 \right) \end{aligned}$$

where *D_c_* is the effective diffusion coefficient on the basic of the concentration; *q_t_* (mmol/g) and *q_e_* (mmol/g) are the amount of adsorbing CO_2_ at a given point of time and at equilibrium stage, respectively; *r* represents the average radius of particle; *b* is the constant; *t* (min) represents the adsorption time; *F* represents the fraction that reaches equilibrium at a given point in time; *k_2_* (mmol/g) and *k_1_* (mmol/g) are the intraparticle diffusion rate constant and interparticle diffusion, respectively; and *B_t_* refers to the mathematical function of *F*; and *n* represents natural number (1,2, 3, to n).

**S1.13 Calculation of the Adsorption Half-life**

The adsorption half-life (t_1/2_) is defined as the time at which the adsorption capacity reaches the equilibrium adsorption capacity (q_e_). The calculation of the half-life is performed by substituting the data into the Avrami model:

$$\begin{aligned} t_{\frac{1}{2}}=\frac{{(ln2)}^{\frac{1}{na}}}{k_{a}} \#\left( S17 \right) \end{aligned}$$

**S1.14 Mouse C****onfined Space Survival Experiment**

This study evaluated the CO_2_ scavenging capabilities of MNRM in a murine confined-space model through two independent experimental batches.

Batch A: Seven-week-old specific pathogen-free (SPF) grade ICR mice (weight 31.0 ± 0.2 g, n=3 per group) were individually placed in a 250 cm³ sealed glass chamber. The blank control group did not contain any CO₂ scavenging material, n=3. The experimental group was placed with 4 g of fully swollen MNRM, n=3. Survival duration was recorded, and lung tissues were subjected to histopathological analysis (H&E staining) post-mortem.

Batch B: Six-week-old SPF-grade ICR mice (body weight: 17.9 ± 0.3 g) were utilized. Similarly, the following experimental groups were set up (n=3 per group): blank control group, MNRM experimental group, and the reference control group with 4 g of commercial MOF (ZIF-8, purchased from Guangdong Advanced Carbon Materials Co., Ltd.). Experiments were conducted in a 250 mL glass reagent bottle with a spout. The probe extension tube of the headspace analyzer (Dansensor® CheckMate3) was inserted into the bottle, and the gas composition inside the bottle was automatically monitored and recorded every 5 minutes.

Note: Due to animal batch updates, there were differences in age and body weight between the two batches of mice, resulting in biological differences in basal metabolic rate (BMR) and CO₂ production rate. Therefore, data from the two batches were analyzed independently. Batch A provided lung tissue section analysis, while batch B provided evidence of gas dynamic changes.

All the animal experiments were approved by the Animal Welfare Committee of Guangxi University (Gxu-2025-243) and were performed according to the guidelines of the Animal Welfare Committee.

**S1.15Lung Tissue Analysis and Hematoxylin–Eosin (HE) Staining**

After the confined space asphyxiation experiment, the lung tissues of the mice were collected, fixed with 4% paraformaldehyde, embedded in paraffin, and sliced into 2.5–4 μm thick sections. For histological analysis, the sections were dewaxed, washed, and stained with hematoxylin and eosin. The lung tissue was then examined for pathological changes. The severity of tissue lesions was classified into five levels: grade 0 (no or very minimal lesions), grade 1 (mild lesions), grade 2 (moderate lesions), grade 3 (severe lesions), and grade 4 (extremely severe lesions).

**S1.16 In vitro cytotoxicity**

Human normal lung epithelial cells (BEAS-2B cells) were used to analyze the biocompatibility of MNRM. The BEAS-2B cells at the logarithmic growth stage were inoculated into a complete medium with 96-well plates according to 7×10^3^ cells/well and cultured overnight in a 5% CO_2_ incubator at 37 °C. The samples were added to the medium for 24 h, the medium was removed, and each well was washed thrice with PBS. Then 10% CCK-8 solution was added into the culture medium according to 100 μL/well, and incubated in a 5% CO_2_ incubator at 37 °C for 2 h. The absorbance value at 450 nm was measured using a microplate reader.

**S2 Supplementary Figures and Tables**


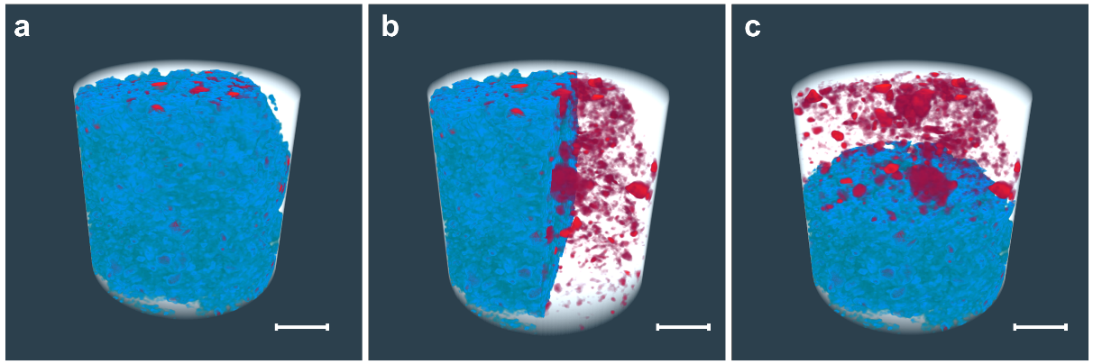


**Fig. S1** Nano-CT characterization of MNRM: **a** The spatial distributions of the high - density (red) and low - density (blue) phases completely overlap. **b** The high - density and low - density phases each occupy half of the space, distributed symmetrically left and right. **c** The high - density and low - density phases each occupy half of the space, distributed symmetrically up and down. (Scale: 100 μm)


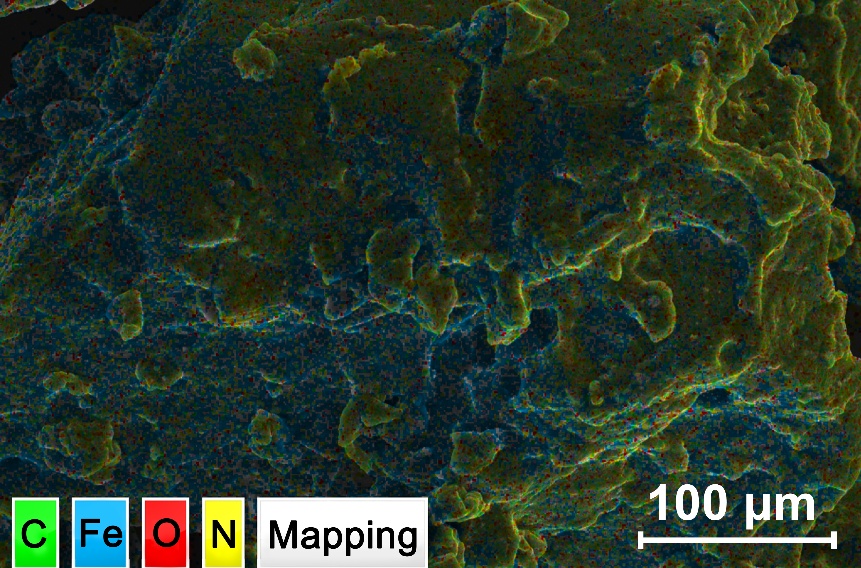


**Fig. S2** SEM-EDS elemental mapping of the MRM


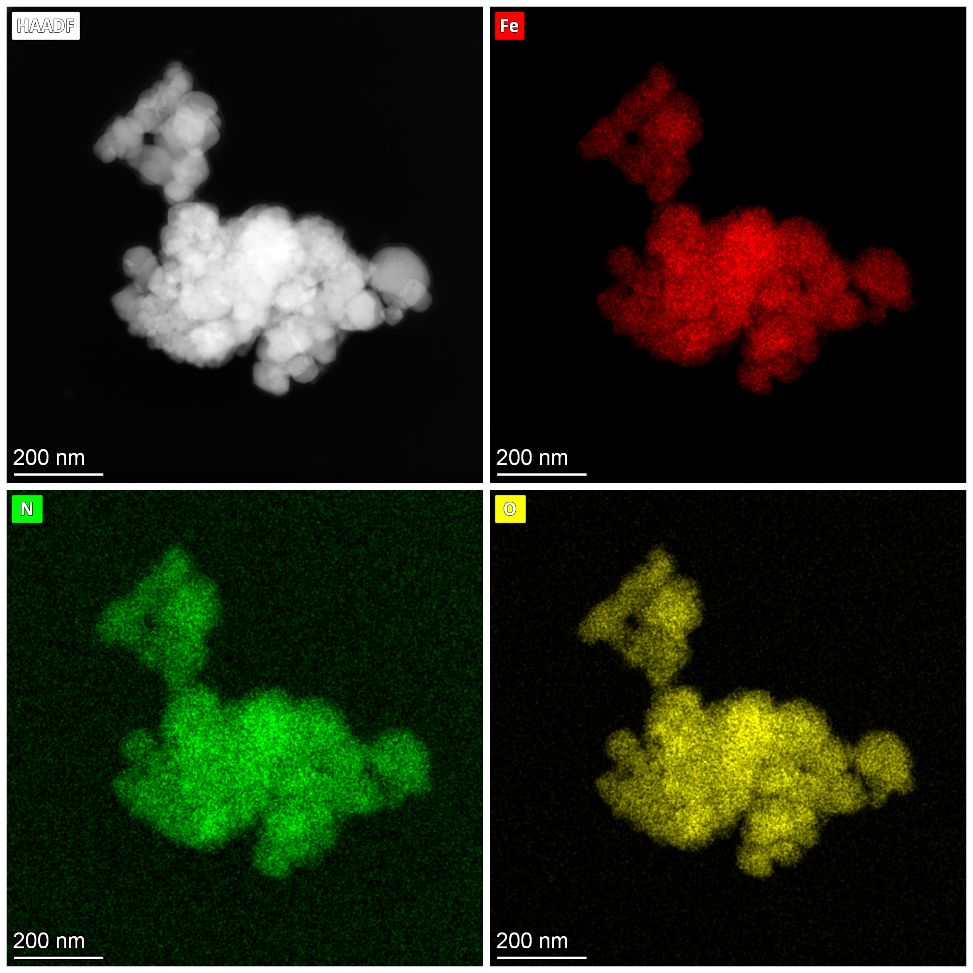


**Fig. S3** TEM and EDS mapping scanning images of aminated Fe_3_O_4_ NPs

**
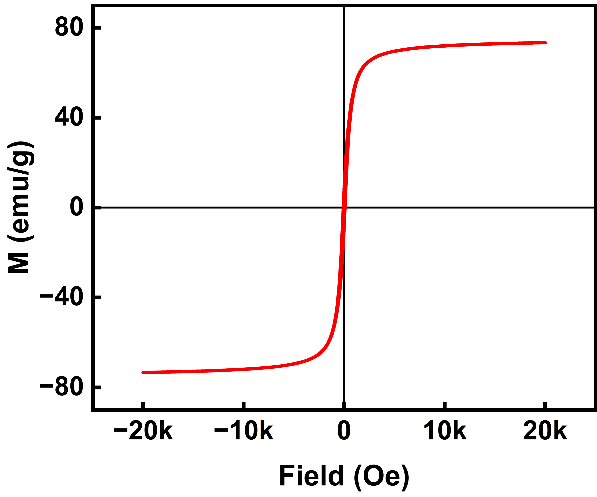
**

**Fig. S4** Magnetic hysteresis curves of Fe_3_O_4_ NPs


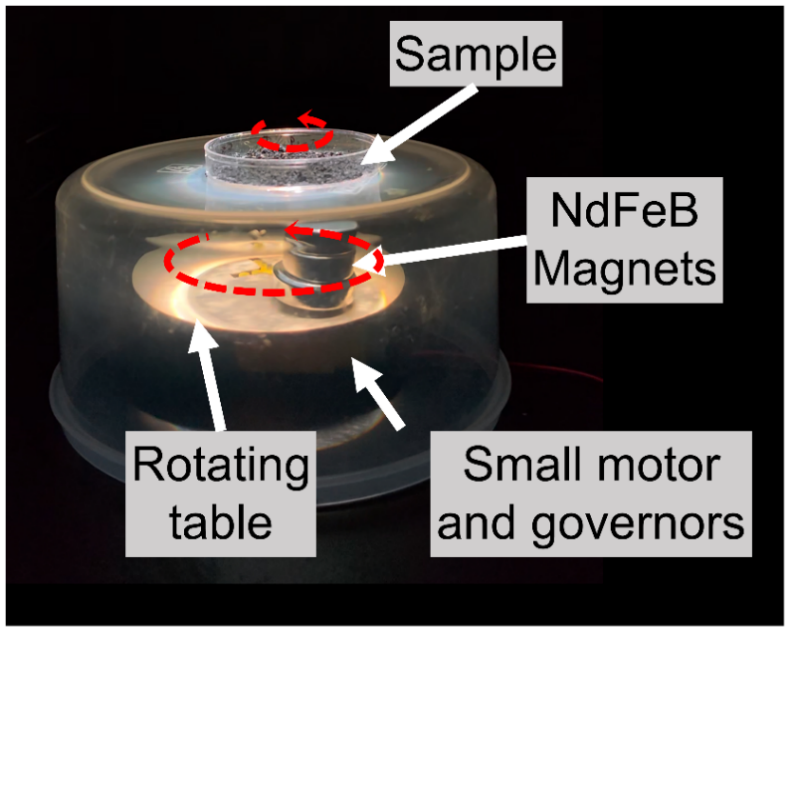


**Fig. S5** Optical image of the experiment of light irradiation and dynamic magnetic field


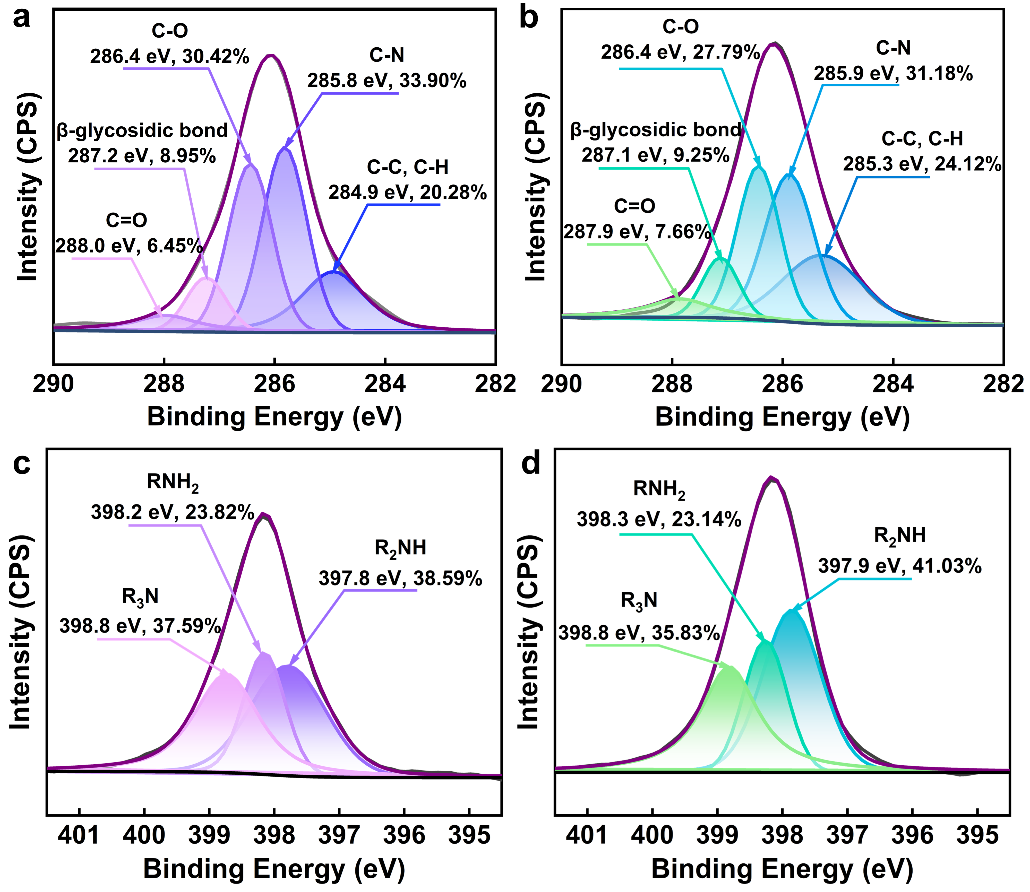


**Fig. S6 a** Deconvoluted C 1s XPS spectra of MNRM. **b** Deconvoluted C 1s XPS spectra of MRM. **c** Deconvoluted N 1s XPS spectra of MNRM. **d** Deconvoluted N 1s XPS spectra of MRM. All data are tested at 25 °C


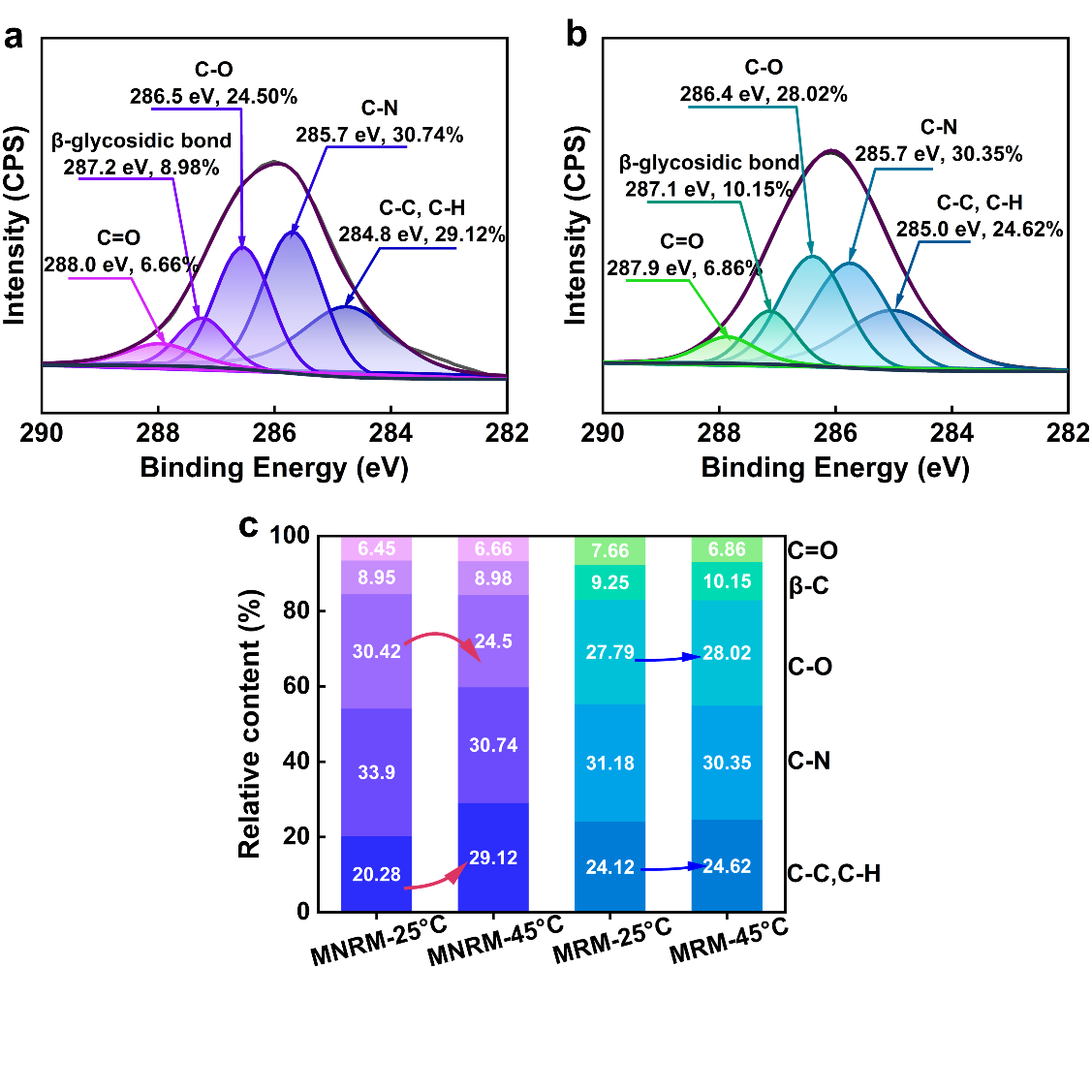


**Fig. S7 a** Deconvoluted C 1s XPS spectra of MNRM at 45 °C. **b** Deconvoluted C 1s XPS spectra of MRM at 45 °C. **c** Contents of carbon-containing groups in MNRM and MRM measured by XPS at 25 °C and 45 °C

**Table S1** Amino Content Determined by EA (C, N and H mode)

|  | **N (%)** | **C (%)** | **H (%)** | **amino content (mmol/g)** |
| --- | --- | --- | --- | --- |
| MNRM | 23.31 | 42.54 | 10.72 | 16.65 |
| MRM | 24.80 | 47.57 | 11.87 | 17.71 |


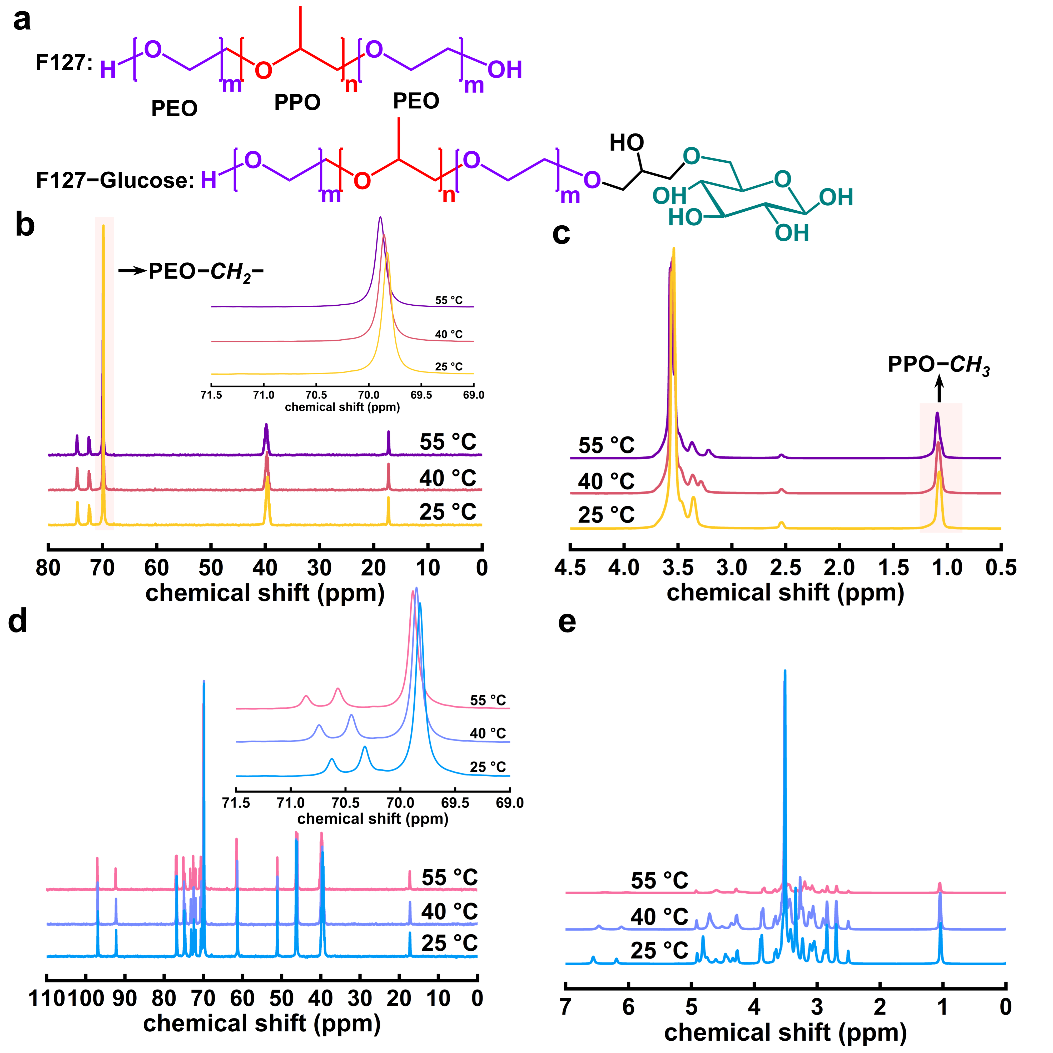


**Fig. S8** **a** Molecular formulae of F127 and F127-grafted Glucose. **b** Liquid ^13^C and **c** ^1^H NMR spectra of F127. **d** Liquid ^13^C and **e** ^1^H NMR spectra of F127- Glucose


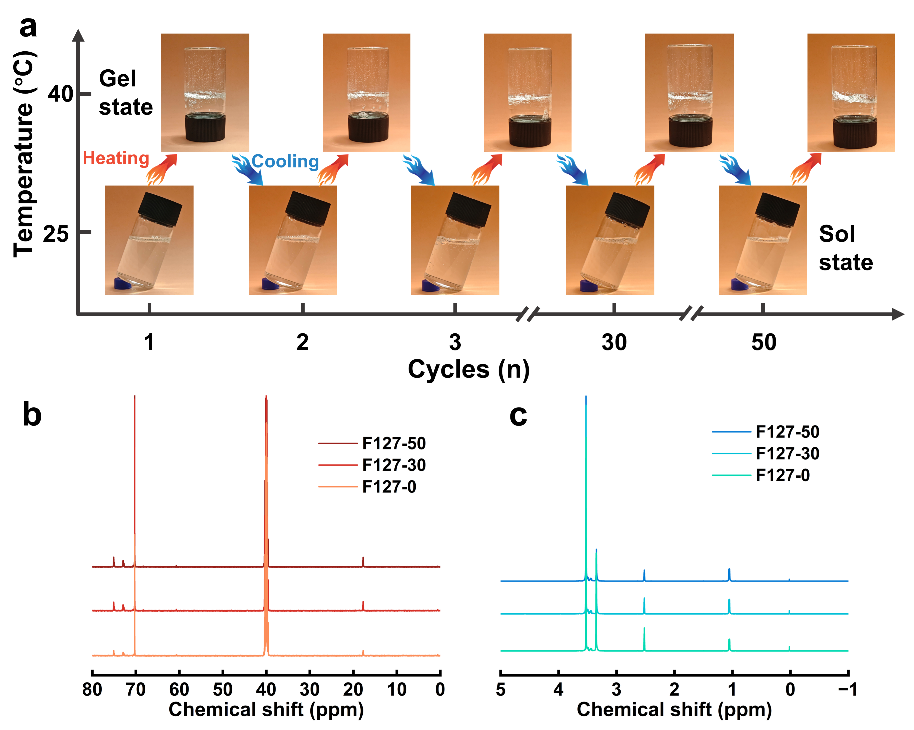


**Fig. S9** **a** Optical photographs of the 50 cyclic changes in the sol state at 25 °C and the gel state at 40 °C of 15wt% F127 aqueous solution. **b** Liquid ^13^C and **c** ^1^H NMR spectra of F127 before variable temperature cycling, after 30 cycles, and after 50 cycles


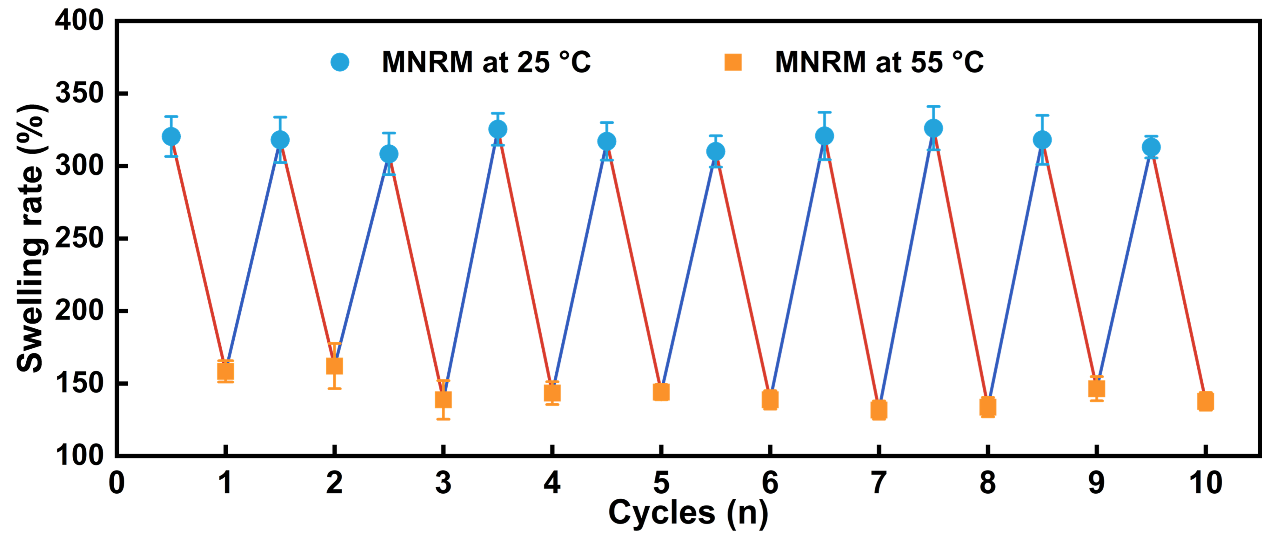


**Fig. S10** Swelling rate of MNRM at alternating temperatures of 25 and 55 °C for 10 cycles


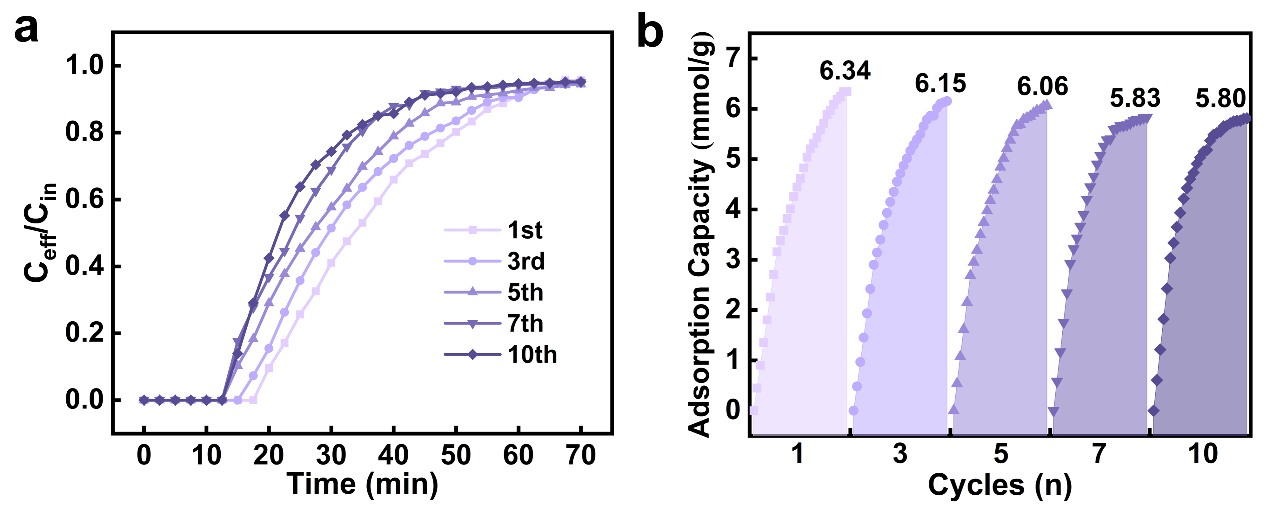


**Fig. S11** **a** Breakthrough curves for CO_2_ adsorption and **b** the corresponding adsorption capacities of MNRM after 180 days of storage (adsorption environment: 25 °C, desorption temperature: 55 °C)

A 15 wt% aqueous solution of F127 exists as a low viscosity sol at 25 °C and undergoes a thermos-reversible transition to a rigid gel state upon heating to 40 °C. Inversion vial tests of F127 with 50 phase transitions confirmed the persistent thermoresponsive switching capability. Furthermore, after 30 and 50 temperature cycles, the ^13^C NMR spectrum and ^1^H NMR spectrum of F127 exhibited no significant chemical shift or peak intensity changes compared to the initial state. This indicates that even after 50 phase transitions, the molecular structure of F127 remains intact.

MNRM demonstrated F127-driven thermo-regulated swelling property, with a high swelling ratio of over 300% at 25 °C and a low swelling ratio of approximately 150% at 55 °C. Crucially, MNRM that had been sealed and stored for at least 180 days underwent 10 regeneration cycles at 55 °C, with the adsorption capacity remaining at 5.80 mmol/g and a regeneration rate of 91.33%, which fully demonstrates its excellent long-term stability and cycle durability.


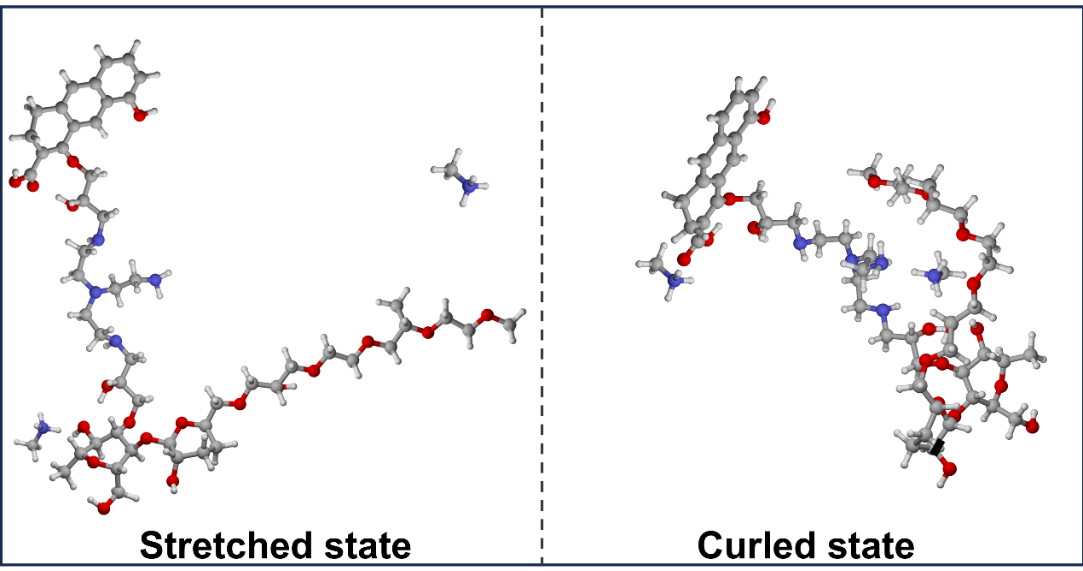


**Fig. S12** Molecular structures for ESP simulations


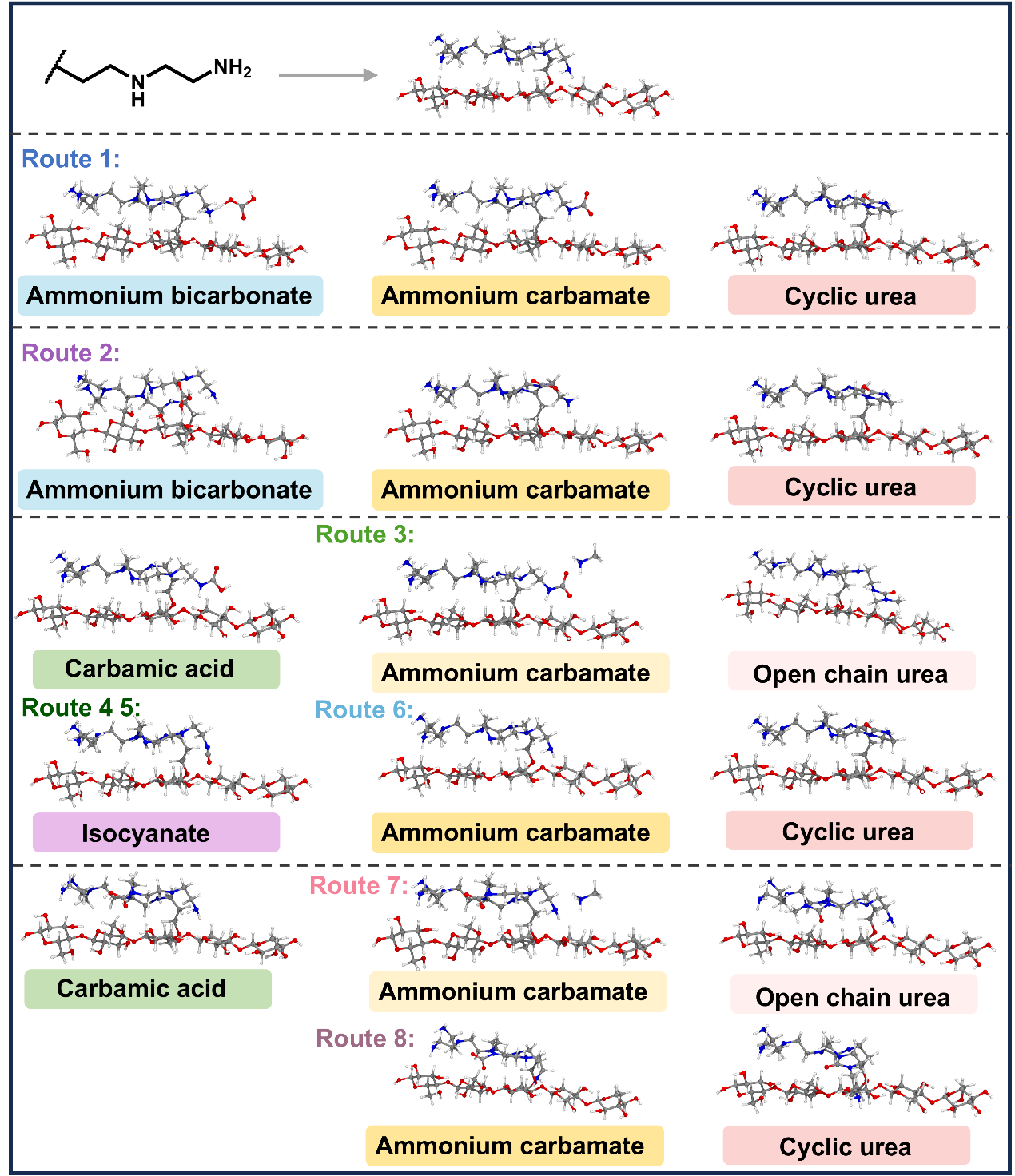


**Fig. S13** Molecular structures for Gibbs free energy calculations


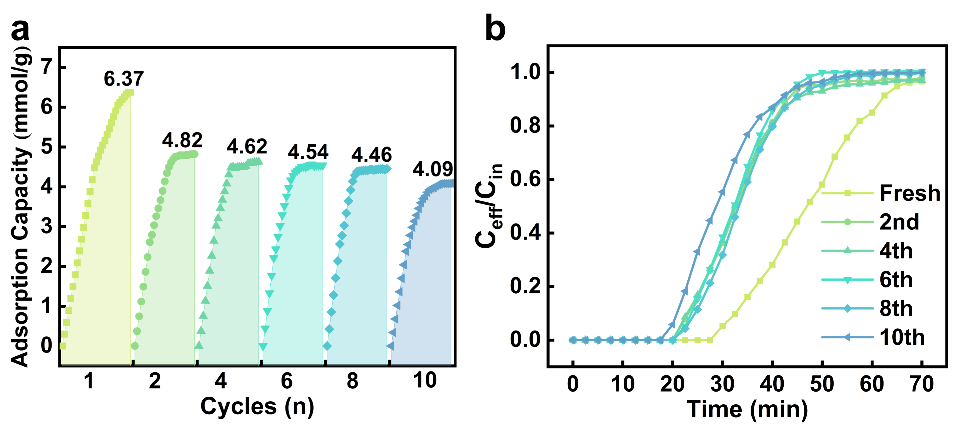


**Fig. S14** **a** Breakthrough curves for CO_2_ adsorption and **b** the corresponding adsorption capacities of MNM (cyclic regeneration condition: external hydrothermal heating at 55 °C)


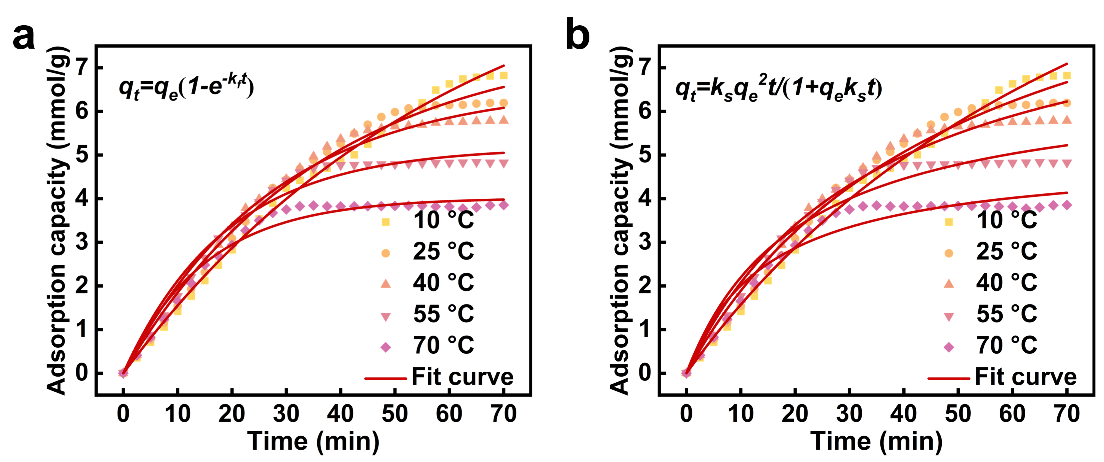


**Fig. S15** **a** Plots of pseudo first-order and **b** pseudo second-order kinetic models for CO_2_ adsorption on MNRM


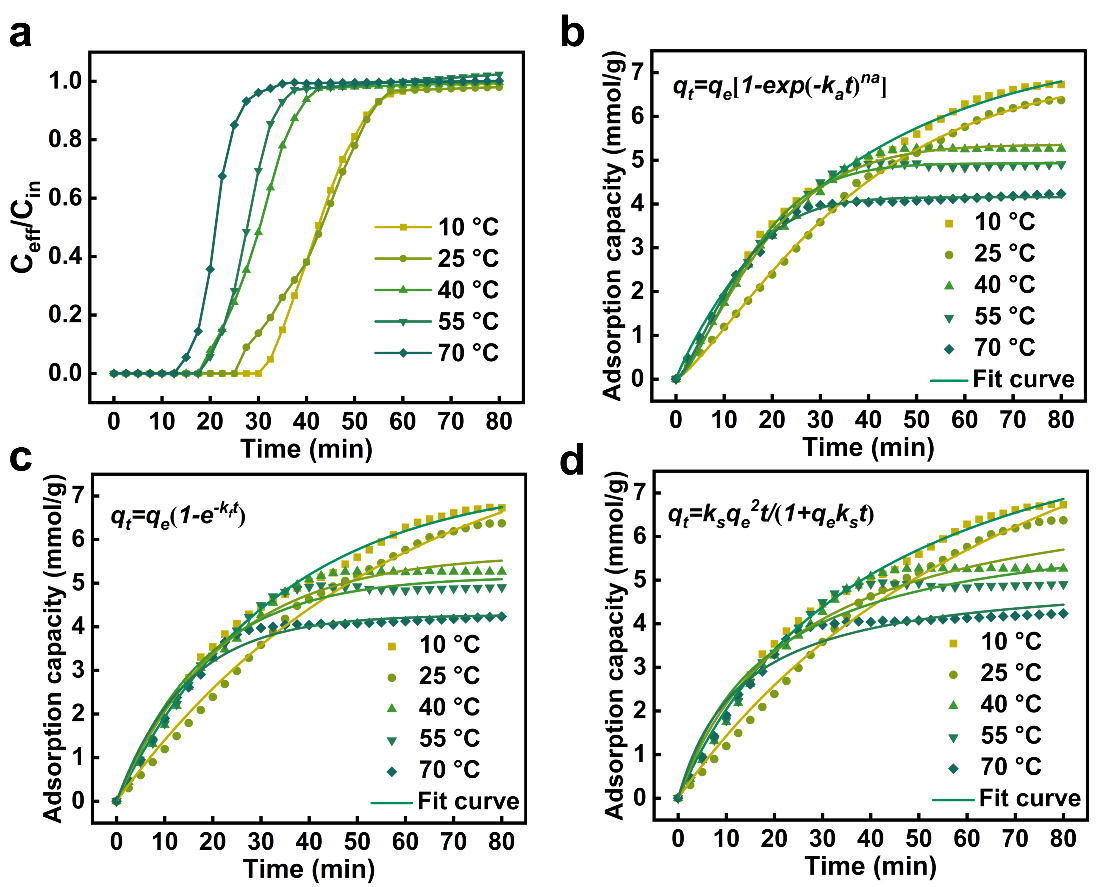


**Fig. S16** **a** Breakthrough curves of MRM at different temperatures. **b** Avrami model, **c** Plots of pseudo first-order and **d** pseudo second-order kinetic models for CO_2_ adsorption on MRM

**Table S2** Kinetic constants of CO_2_ adsorption on the adsorbents at different temperatures


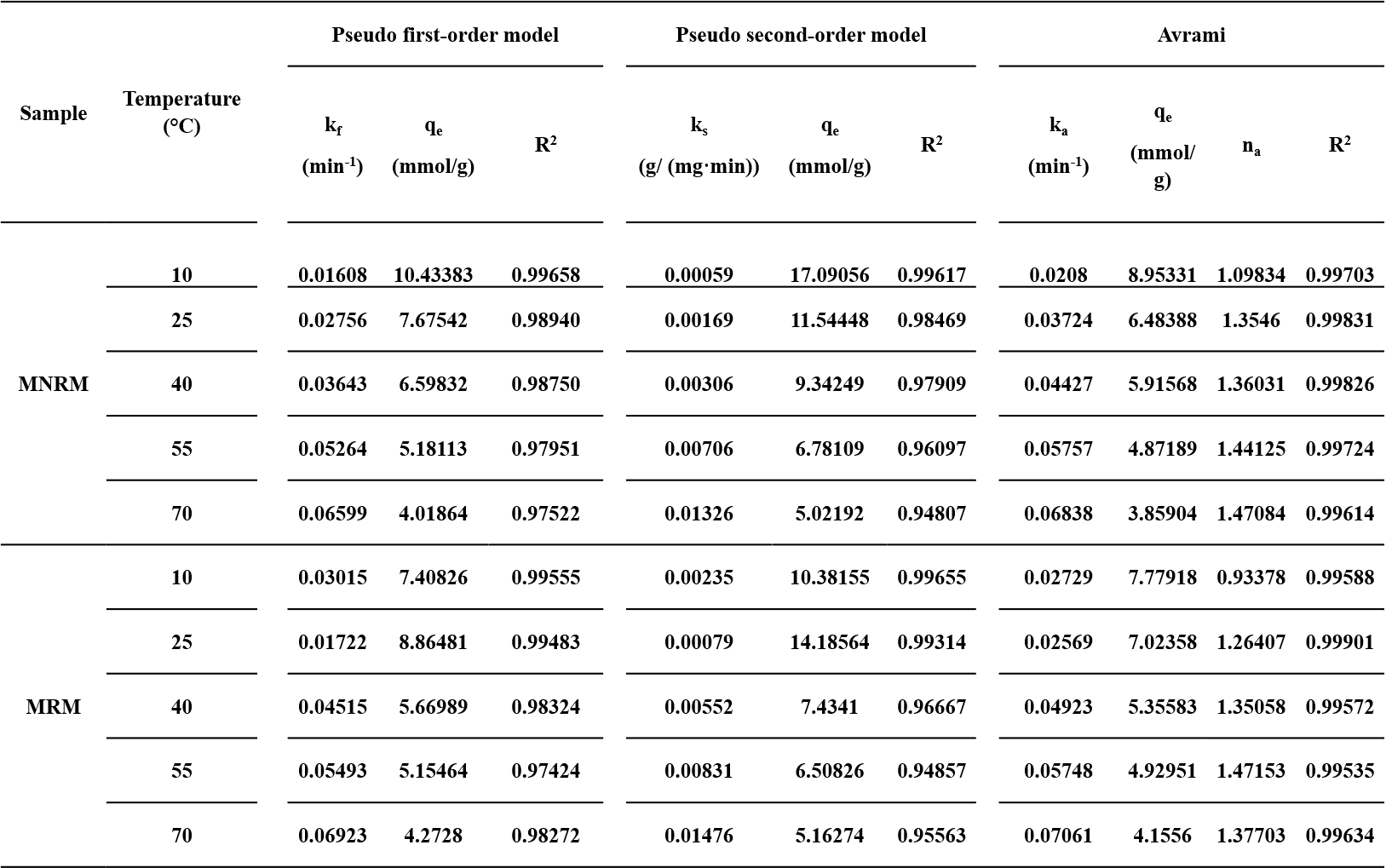


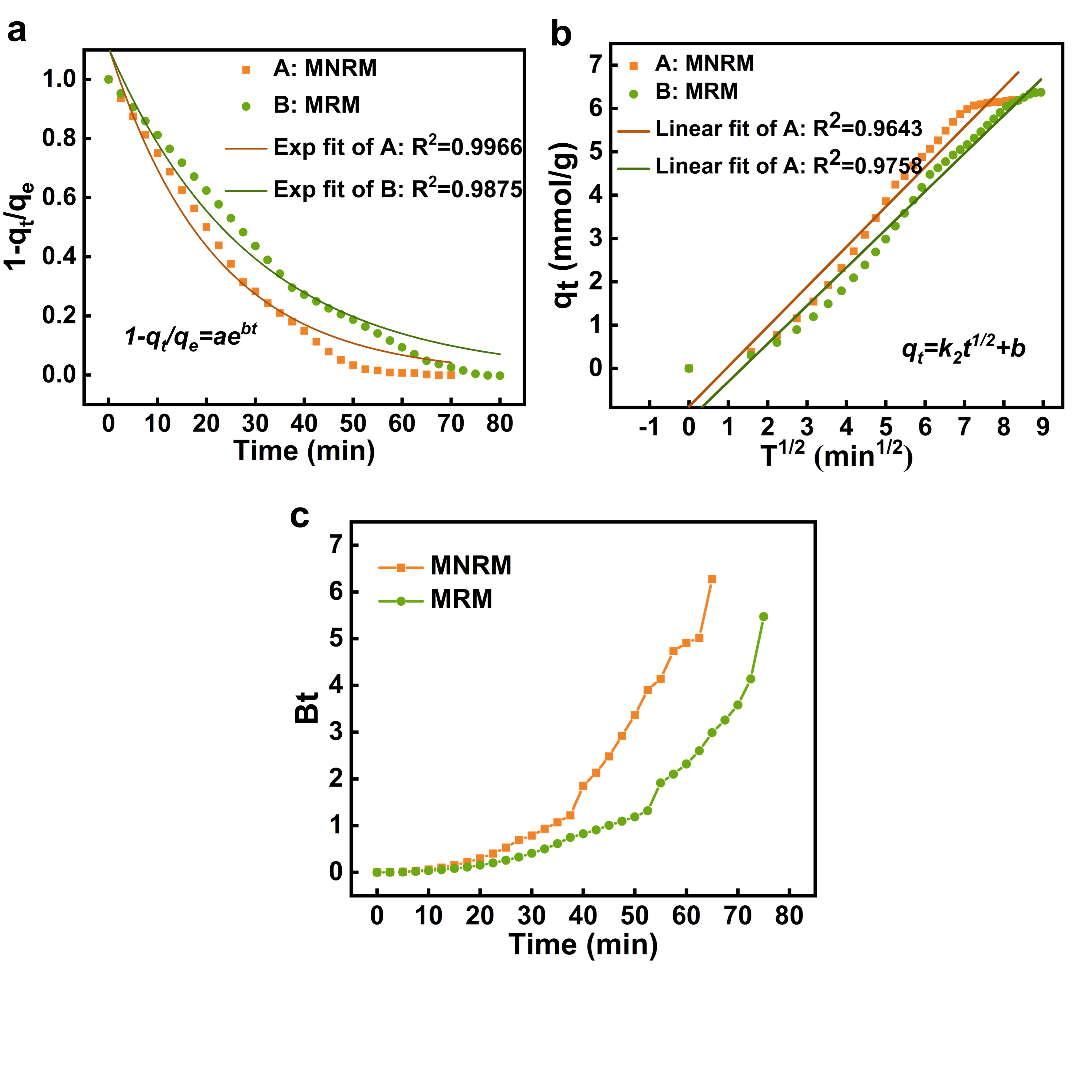


**Fig. S17** Plots of diffusion models for CO_2_ adsorption on MNRM and MRM. **a**Non-linear fitting of interparticle diffusion model, **b** linear fitting of intraparticle diffusion model, and **c** data for Boyd’s film diffusion model with Bt plotted as a function of time


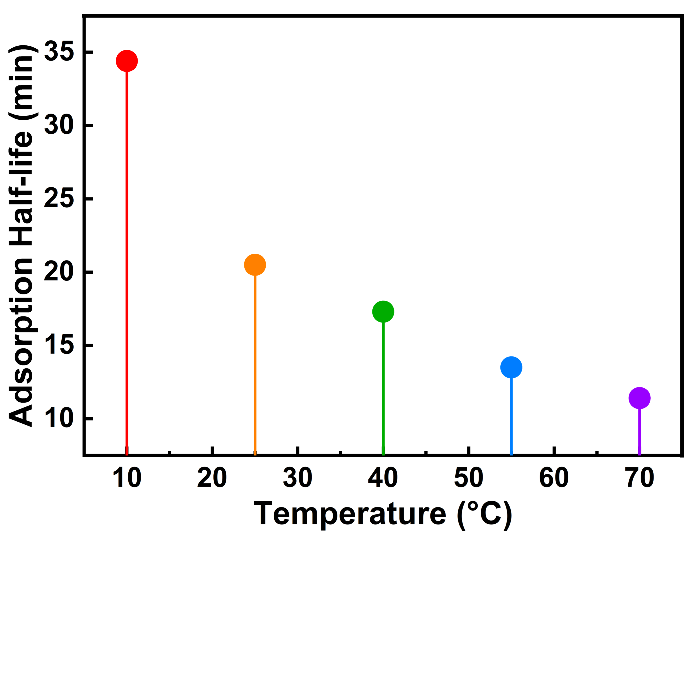


**Fig. S18** Adsorption half-lives at different temperatures calculated from the Avrami model


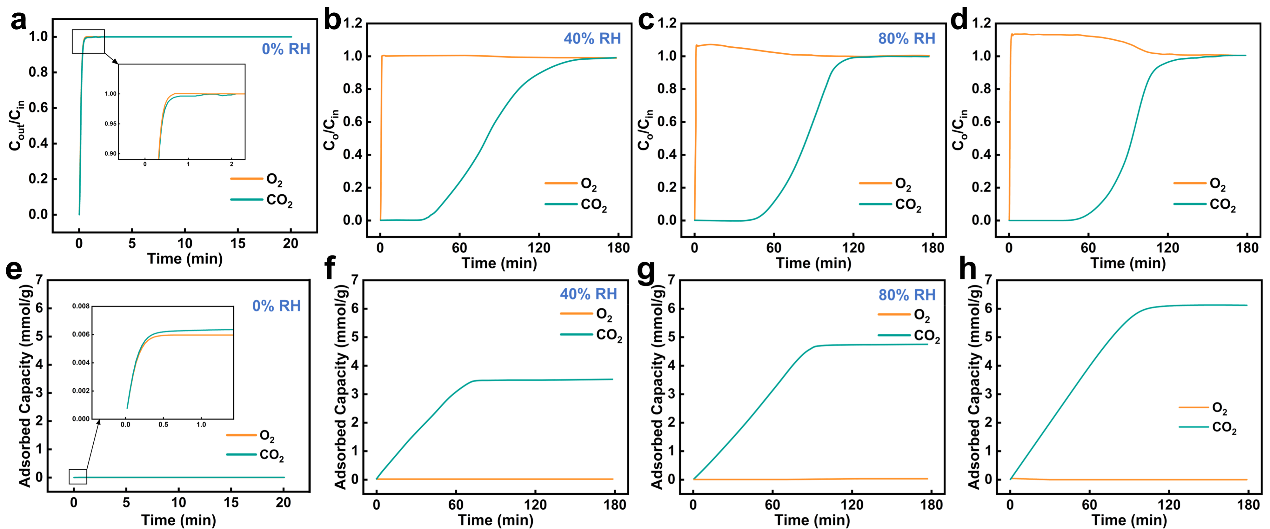


**Fig. S19** Breakthrough curves (**a**-**c**) and corresponding adsorption capacities (**e-g**) of multi-component gas competitive adsorption of dry MNRM under different humidity conditions. Breakthrough curves **d** and corresponding adsorption capacities **h** of fully swollen MNRM. (The multi-component gas consists of 10 v/v% CO_2_, 10 v/v% O_2_ and 80 v/v% N_2_. Test temperature: 25 °C )

A multi-component gas analyzer was used to characterize the selective adsorption properties of MNRM for CO₂ and O₂ under different humidity conditions. The breakthrough curves indicate that regardless of the initial state of MNRM (dry or wet) or changes in the relative humidity (RH) of the mixed gas, MNRM exhibits extremely low adsorption affinity for O₂. In sharp contrast, the adsorption capacity of MNRM for CO₂ shows a significant increase trend as gas humidity rises. Specifically, under 0% RH, 40% RH, and 80% RH conditions, the adsorption capacities were 0.006 mmol/g, 3.58 mmol/g, and 4.88 mmol/g, respectively. After complete swelling, MNRM achieved a high CO₂ adsorption capacity of 6.12 mmol/g in a CO₂/O₂/N₂ mixed gas system. The above results indicate that, unlike MOF materials whose adsorption performance is susceptible to water, moisture exhibits a clear promotional effect on MNRM's CO₂ adsorption, consistent with the analysis conclusions in Section 3.3 of the manuscript, “CO₂ Adsorption Performance of MNRM at Wet and Dry State”.

**
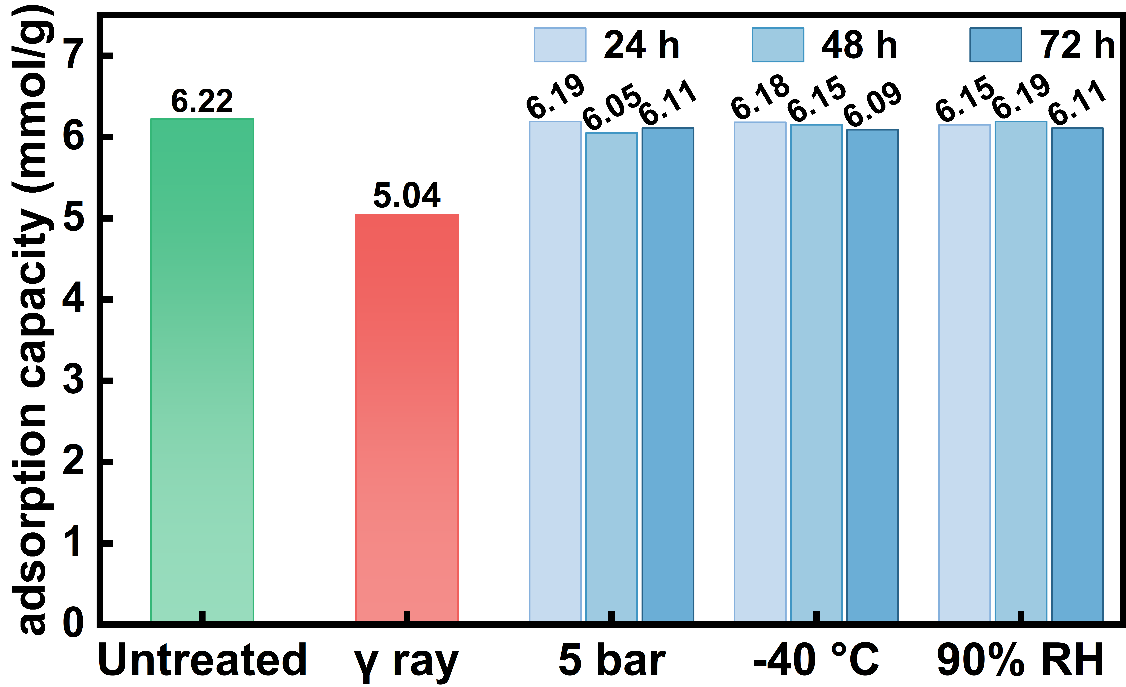
**

**Fig. S20** The adsorption capacity of MNRM before and after pretreatment with radiation (dose rate of 0.837 kGy/h), high pressure (5 bar), low temperature (-40 °C), and high humidity (90% RH)

To assess the stability of MNRM's performance under extreme conditions, four pre-treatment conditions were set: radiation, high pressure, low temperature, and high humidity. The results showed that after irradiation with γ-rays at a dose rate of 0.837 kGy/h, the CO₂ adsorption capacity of MNRM decreased from the initial 6.22 mmol/g to 5.04 mmol/g, but still retained 81.03% of its initial capacity. This may be due to the generation of free radicals during irradiation, which induced the oxidation of amino groups in MNRM, thereby weakening its adsorption capacity. After 24 h, 48 h, and 72 h of pretreatment under conditions of 5 bar, -40 °C, and 90% RH, respectively, MNRM demonstrated excellent tolerance, with its CO₂ adsorption performance remaining stable and no significant decline observed. In summary, MNRM demonstrates good performance stability under simulated extreme conditions, proving its potential application value under various harsh conditions.


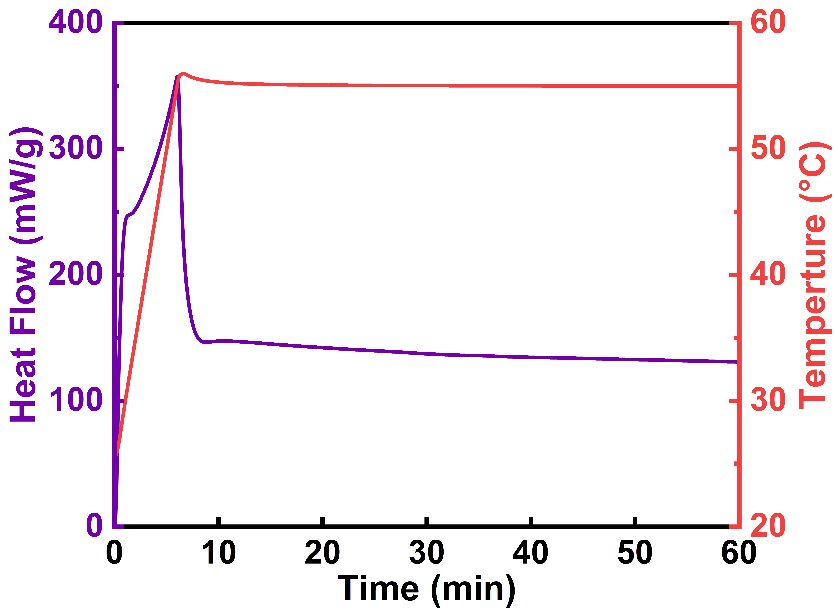


**Fig. S21** Differential scanning calorimetry (DSC) data collected for MNRM under flowing N_2_

The regeneration energy of MNRM consists of two components: (1) the sensible heat required to heat MNRM from 25 °C to 55 °C, and (2) the enthalpy of desorption corresponding to the removal of CO₂ and water during a 50-minute hold at 55 °C. Integrating the DSC data, the estimated regeneration energy consumption of MNRM was 314.86 J/g. It should be noted that this energy consumption estimate is a lower limit. The actual regeneration process may be affected by a variety of factors, such as the heating efficiency of the equipment. Meanwhile, MNRM can also efficiently utilize solar energy to achieve desorption through its own light-to-heat conversion. Therefore, in terms of photothermal desorption, the energy consumption of MNRM regeneration can be considered to be significantly reduced, because solar energy is a renewable clean energy source that can greatly reduce dependence on traditional energy sources. This characteristic gives MNRM a great advantage in terms of sustainable development and environmental protection applications.


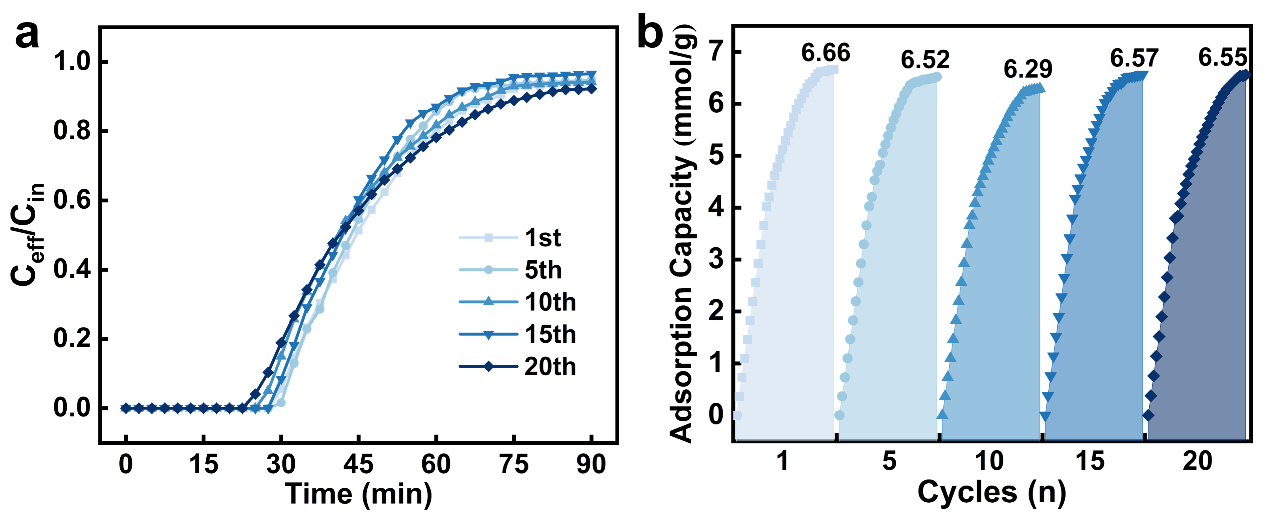


**Fig. S22** **a** Breakthrough curves for CO_2_ adsorption and **b** the corresponding adsorption capacities of MNRM that adsorbed at 15 °C and desorbed at 55 °C to simulate the day-night cycle of low-temperature regeneration

Regarding the validation of regeneration performance under day-night temperature difference cycles, given the excellent adsorption kinetics of MNRM (which reaches saturation within 70 minutes), the traditional 24-hour day-night cycle was compressed into an accelerated cycle consisting of 90 min of adsorption at 15 °C (to simulate the low temperature of nighttime) and 90 min of desorption at 55 °C. The results show that, compared with the adsorption capacity of 6.19 mmol/g measured under normal temperature (25 °C) conditions (Fig 6 **d** and **g**), MNRM exhibits stronger adsorption capacity under simulated nighttime temperatures of 15 °C, with adsorption capacity increasing to 6.66 mmol/g, and also achieves stable regeneration capacity at 55 °C.


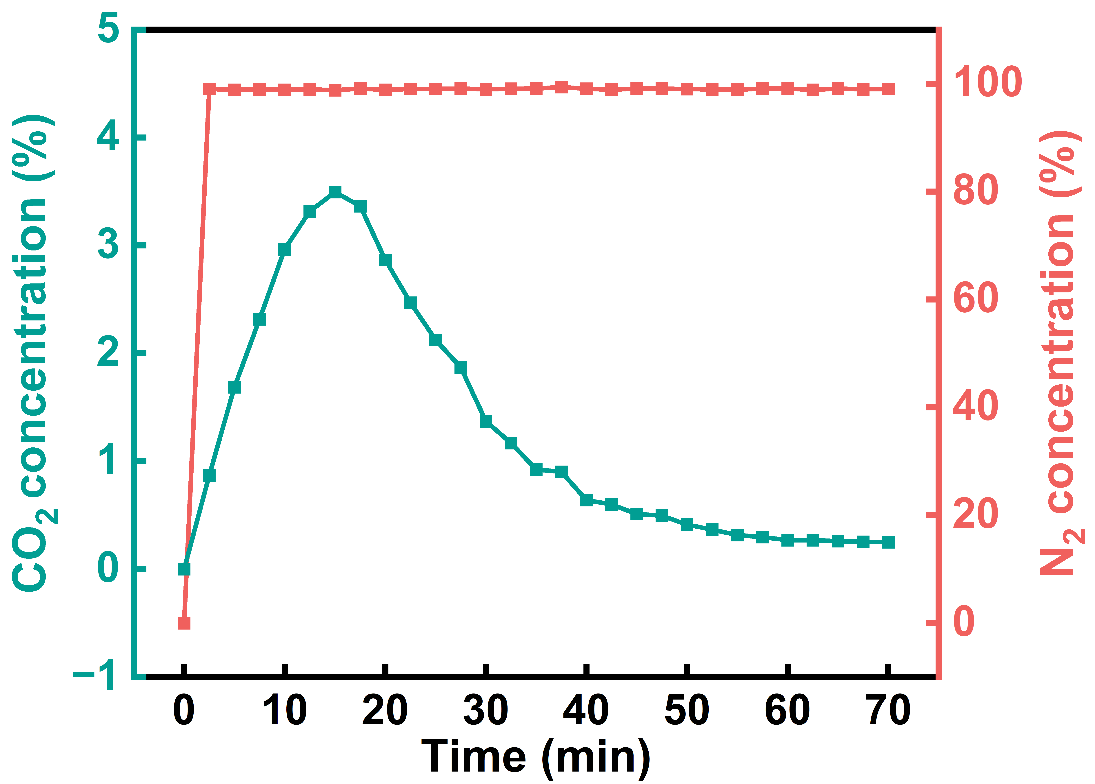


**Fig. S23** Gases generated during the regeneration process, where N_2_ is the purge carrier gas

Potential Challenges and Solutions: The present design of MNRM is principally based on laboratory ideal conditions. In the course of transitioning to large-scale production and engineering applications, the core challenges pertain to cost control and long-term cycle stability, which must be capable of withstanding thousands to tens of thousands of cycles. In order to optimize costs, a strategy for the utilization of low-cost raw materials will be adopted, such as using sugarcane bagasse pulp and industrial-grade magnetite powder (Fe₃O₄ content 80 ~ 90%). With regard to cycle stability, laboratory tests have demonstrated the capacity of MNRM to function reliably over dozens of cycles. The experimental data obtained provide a significant foundation for the development of models and the prediction of long-term cycle decay behaviour on a scale ranging from thousands to tens of thousands of cycles.


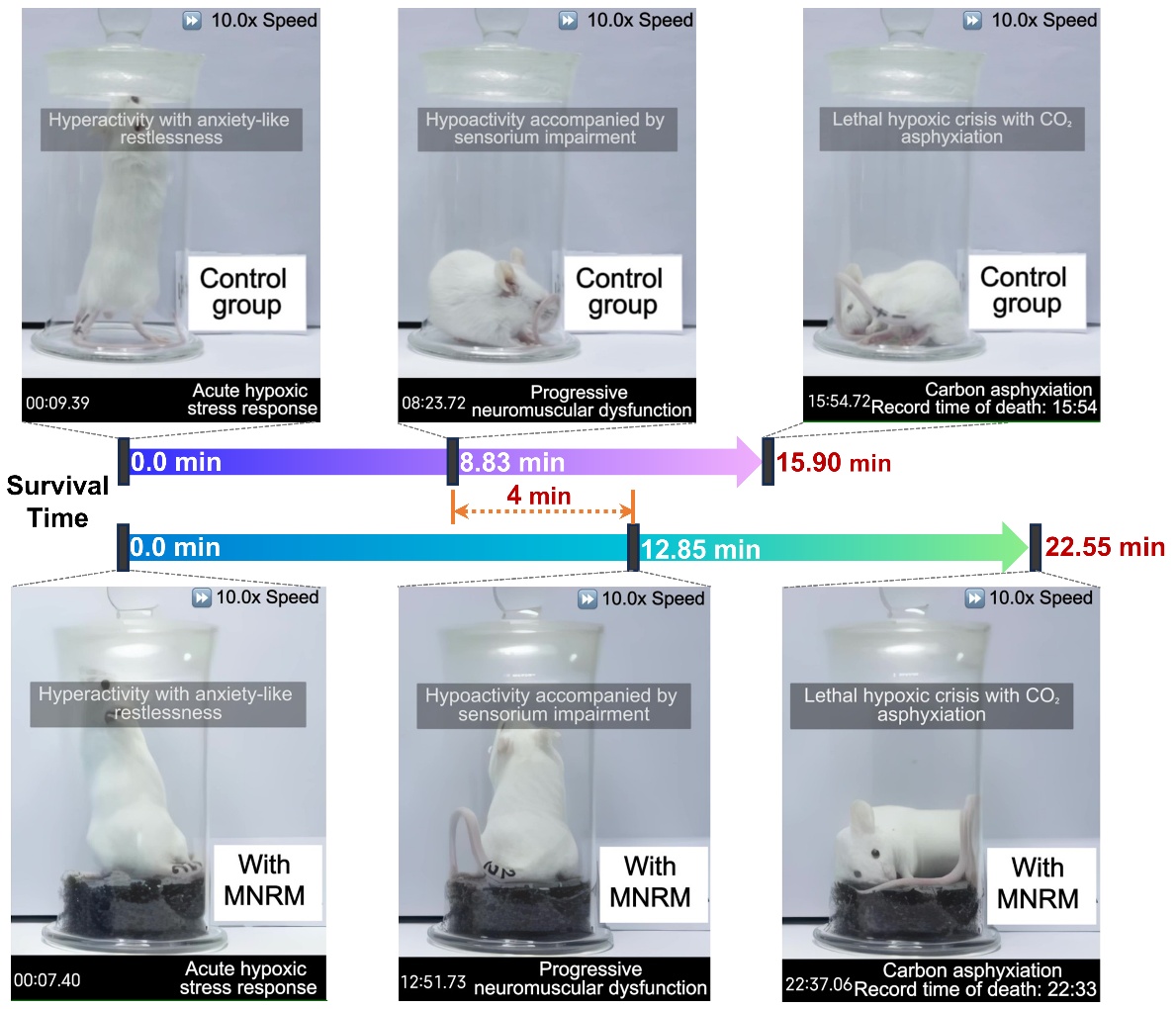


**Fig. S24** Optical photographs of control group and MNRM experimental group mice of batch A at different stages


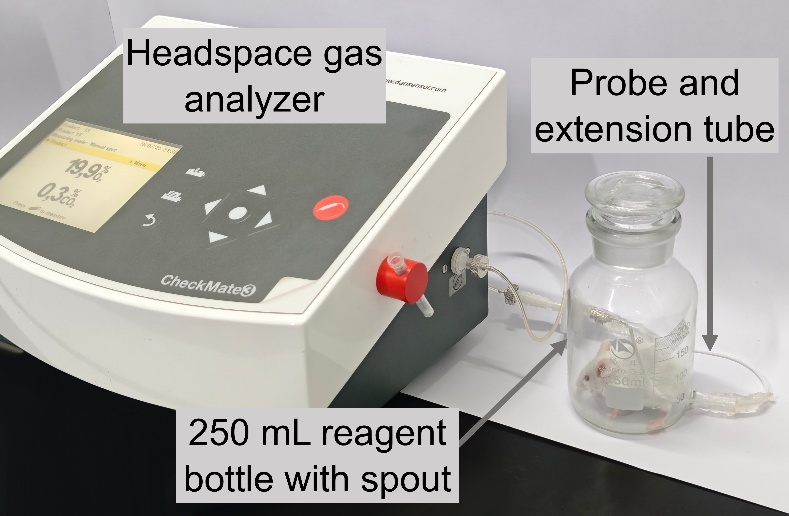


**Fig. S25** Optical photograph of device for confined space survival experiment of batch B mice


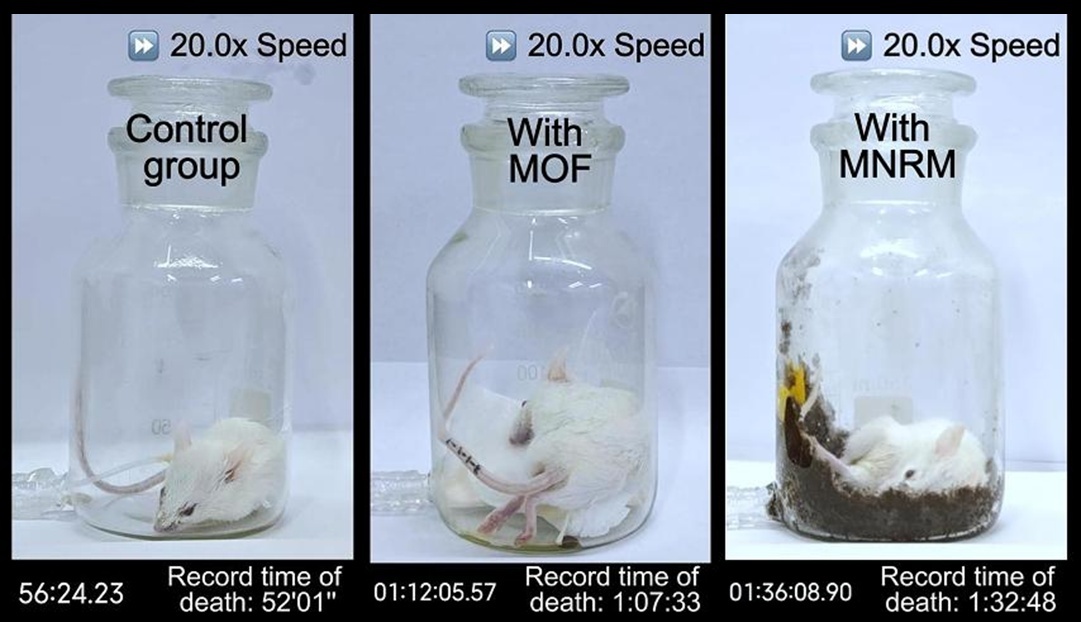


**Fig. S26** Optical photographs of mice in control group, MOF reference control group and MNRM experimental group of batch B


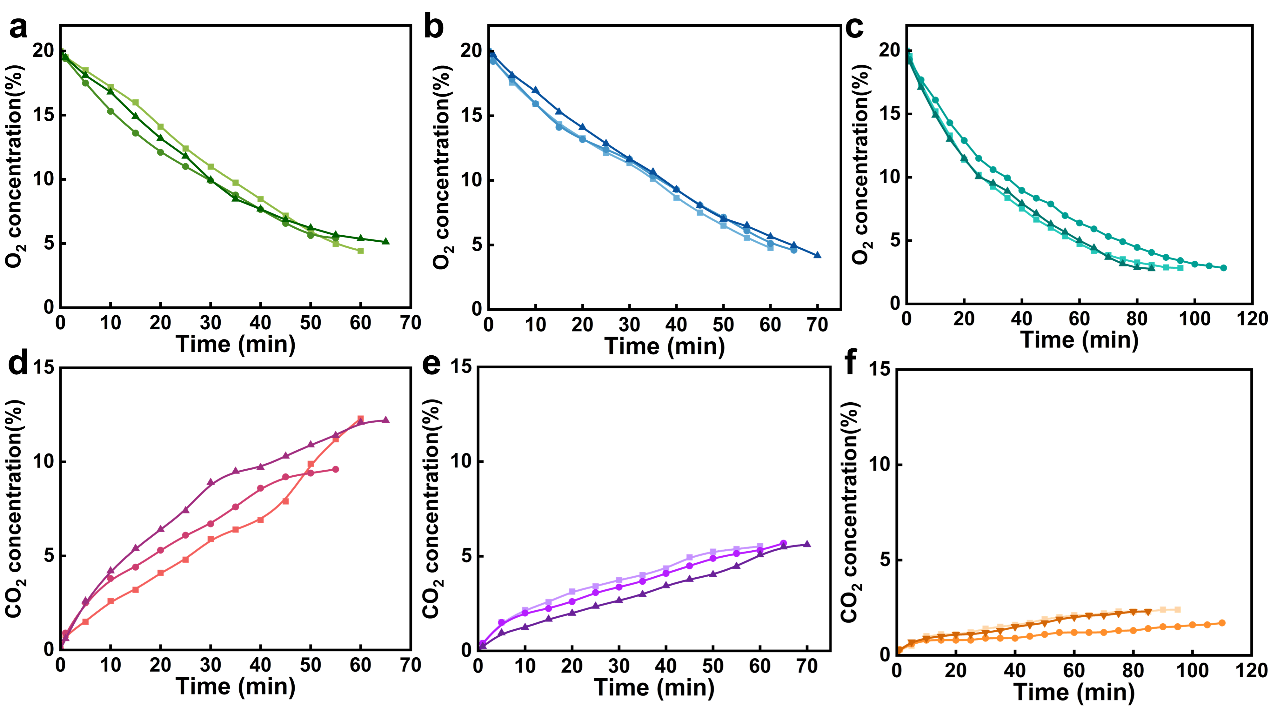


**Fig. S****27** Real-time O_2_ and CO_2_ concentration curves in sealed containers for mice in the blank control group (**a** and **d**), MOF reference control group (**b** and **e**) and MNRM experimental group (**c** and **f**) of batch B


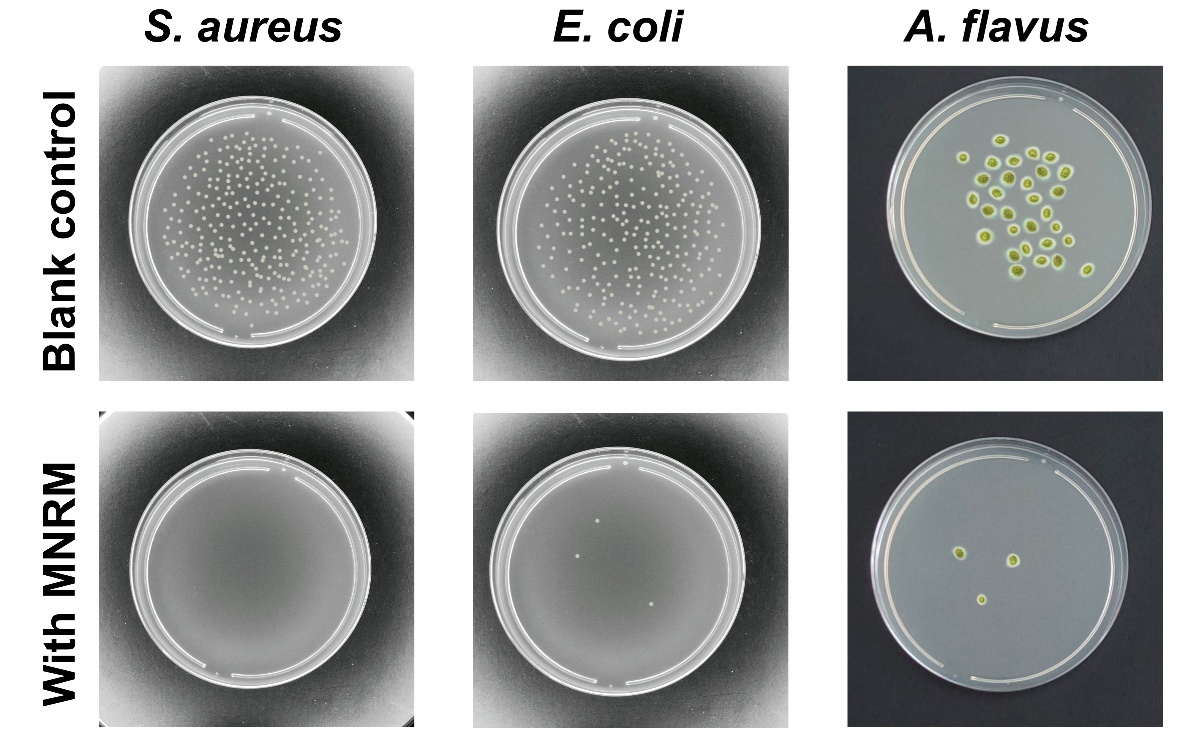


**Fig. S28** Antibacterial effect of blank control group and experimental group with MNRM against *Staphylococcus aureus* (*S. aureus*), *Escherichia coli* (*E. coli*), and *Aspergillus flavu***s** (*A. flavus*).

**Supplementary References**

1. W. Lu, X. Shi, H. Zhou, W. Luo, L. Wang et al., Tailoring and properties of a novel solar energy-triggered regenerative bionic fiber adsorbent for CO_2_ capture. Chem. Eng. J. **449**, 137885 (2022). <https://doi.org/10.1016/j.cej.2022.137885>
2. X. Duan, X. Song, R. Shi, X. Wang, S. Chen et al., A theoretical perspective on the structure and thermodynamics of secondary organic aerosols from toluene: molecular hierarchical synergistic effects. Environ. Sci.: Nano **9**(3), 1052–1063 (2022). <https://doi.org/10.1039/D1EN00959A>
3. X. Song, H. Guo, J. Tao, S. Zhao, X. Han et al., Encapsulation of single-walled carbon nanotubes with asymmetric pyrenyl-gemini surfactants. Chem. Eng. Sci. **187**, 406–414 (2018). <https://doi.org/10.1016/j.ces.2018.05.009>
4. S. Grimme, S. Ehrlich, L. Goerigk, Effect of the damping function in dispersion corrected density functional theory. J. Comput. Chem. **32**(7), 1456–1465 (2011). <https://doi.org/10.1002/jcc.21759>
5. T. Lu, F. Chen, Multiwfn: a multifunctional wavefunction analyzer. J. Comput. Chem. **33**(5), 580–592 (2012). <https://doi.org/10.1002/jcc.22885>
6. C. Bannwarth, S. Ehlert, S. Grimme, GFN_2-_*_x_*TB: an accurate and broadly parametrized self-consistent tight-binding quantum chemical method with multipole electrostatics and density-dependent dispersion contributions. J. Chem. Theory Comput. **15**(3), 1652–1671 (2019). <https://doi.org/10.1021/acs.jctc.8b01176>
7. P. Pracht, F. Bohle, S. Grimme, Automated exploration of the low-energy chemical space with fast quantum chemical methods. Phys. Chem. Chem. Phys. **22**(14), 7169–7192 (2020). <https://doi.org/10.1039/C9CP06869D>
8. S. Grimme, S. Ehrlich, L. Goerigk, Effect of the damping function in dispersion corrected density functional theory. J. Comput. Chem. **32**(7), 1456–1465 (2011). <https://doi.org/10.1002/jcc.21759>
